# The aggressive colorectal cancer subtype marker HTR2B has a dual role depending on the tumor microenvironment

**DOI:** 10.1186/s12964-025-02395-6

**Published:** 2025-10-01

**Authors:** Idan Carmi, Adrián Orosz, Szabolcs Hajdó, Anikó Zeöld, Tamás Hegedűs, Dóra Kelemen-Győri, Julianna Pozsár, Tamás Tölgyes, Zoltán Wiener

**Affiliations:** 1 Department of Genetics, Cell- and Immunobiology, Semmelweis University, Budapest, Hungary; 2 Department of Biophysics and Radiation Biology, Semmelweis University, Budapest, Hungary; 3 Biophysical Virology Research Group, HUN-REN-SU, Budapest, Hungary; 4 Department of Pathology, Uzsoki Teaching Hospital, Budapest, Hungary; 5 Department of General Surgery and Surgical Oncology, Uzsoki Teaching Hospital, Budapest, Hungary

**Keywords:** Colorectal cancer, Collagen, Organoid, Consensus molecular subtype, Notch, 5-HT, 5-hydroxytryptamine, CMS4

## Abstract

Colorectal cancer (CRC) is the second-third most frequent cancer type in the Western countries, with significant inter- and intra-patient cellular heterogeneity. The transcriptomics-based CRC classification identified a cluster of patients with epithelial-mesenchymal transition (EMT), the accumulation of fibroblasts, and dismal prognosis. Although the serotonin receptor HTR2B is thought to be one of the epithelial markers of this aggressive CMS4 subgroup, its precise role in CRC tumorigenesis is still largely unknown. By using patient-derived organoids (PDO), we prove the heterogeneous expression of this receptor that is under the control of the mTOR pathway. Unfavorable conditions, applying the clinically used 5-fluorouracil, co-culturing with fibroblasts, and collagen-I accumulation in the extracellular matrix (ECM) increased the number of HTR2B + cells. Interestingly, whereas stimulating HTR2B had no effect on PDOs under normal conditions, it reduced cellular survival under unfavorable conditions when carbohydrates were lacking. In contrast, serotonin and an HTR2B agonist induced invasion of HTR2B + tumor cells only in collagen-I, representing a permissive ECM for cellular migration. CRC cells positive for HTR2B also expressed the NOTCH3 receptor and NOTCH target genes at a higher intensity than cells with low HTR2B levels. Similar to HTR2B + organoids, NOTCH3 + PDOs had increased EMT markers and HTR2B levels. Furthermore, blocking NOTCH activity inhibited the serotonin-induced invasion. Collectively, our results show that the serotonin receptor HTR2B marks a CRC cell population with increased proliferative capacity, invasive potential, and partial EMT, and they highlight the critical role of ECM in shaping the effect of serotonin in CRC.

**Graphical Abstract:**

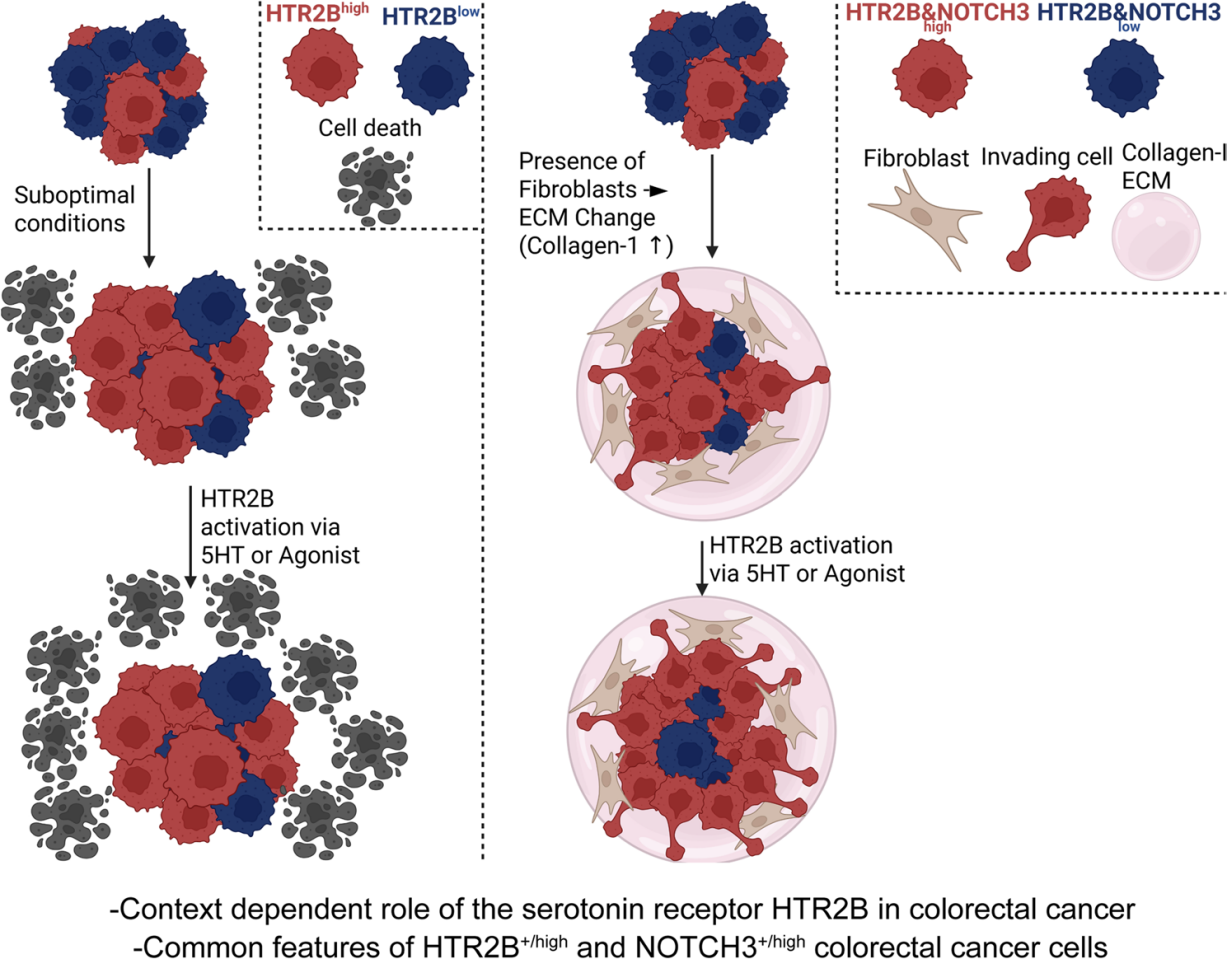

**Supplementary Information:**

The online version contains supplementary material available at 10.1186/s12964-025-02395-6.

## Introduction

Colorectal cancer (CRC) is a heterogeneous disease both genetically and phenotypically. In the majority of CRC patients, the WNT pathway is abnormally over-regulated either by mutations or epigenetic changes. One of the most frequent mutations occurs in the APC gene, resulting in the ligand-independent stimulation of this pathway. Recently, the transcriptomics-based consensus molecular subtyping (CMS) of CRC has been set up where CMS1-4 subgroups have different features [1]. Of these four groups, CMS4 with stromal infiltration and epithelial-mesenchymal transition (EMT) has the worst prognosis with no effective cure. Although samples for CMS classification had been produced from whole tumors, further studies indicated that CRC cell lines can also be categorized into these subgroups [2].

The accumulation of cancer-associated fibroblasts (CAFs), an essential and abundant cell type in the stroma, results in worse patient survival in CRC [3]. A recent organoid-stroma biobank suggested that fibroblasts can only partially shift organoid phenotype towards the CMS4 group [4]. CAFs are also a major source of extracellular matrix (ECM) proteins, and they are important cells in ECM remodeling, inducing invasion as a critical step toward metastasis formation. Interestingly, the accumulation of collagen-I in the ECM inversely correlates with patient survival [5]. Furthermore, AKT-dependent NOTCH3 activation was shown to drive tumor progression in a mouse model of stroma-rich CRC [6]. However, other studies shed light on the complex role of the NOTCH pathway in CRC tumorigenesis. Whereas inhibiting NOTCH directly after inducing APC mutation reduced tumor number, this effect disappeared later in tumorigenesis [7].

Since CMS subgrouping is based on gene expression patterns, its clinical application is difficult. Thus, several studies have aimed to develop methods to simplify patient classification into CMS groups. Recently, a mini-classifier based on the expression of four genes has been developed [8], and an immunohistochemistry-based protocol has also been suggested with CDX2, FRMD6, ZEB1, and HTR2B markers [9]. Out of these molecules, HTR2B encodes one of the serotonin receptors, whose role remains unclear in CRC to date.

Serotonin (5-hydroxytryptamine, 5-HT) has a dual role in CRC development, largely depending on the applied model, the genetic background, and the tumor progression stage. While it inhibits tumorigenesis in chemically induced CRCs [10], many studies found the tumor-promoting role of this biogenic amine in the later stages of cancer development [11, 12]. By using cell cultures and xenograft mouse models, recent studies found that inhibition of HTR2B-mediated serotonin signaling suppressed tumor growth in CRC [13], and that HTR2B facilitated metastasis [14]. In pancreas ductal adenocarcinoma (PDAC), tumor cells were identified as a major source of serotonin, and this molecule promoted tumorigenesis in unfavorable conditions [15]. Thus, the effect of serotonin may depend not only on the cancer type but also on the applied model system. In addition, the regulation of HTR2B expression, the more detailed phenotype of cells with HTR2B expression, and the effect of fibroblasts and ECM composition on HTR2B level and functions are largely unknown in CRC.

Interestingly, CMS classification in mice identified major differences compared to humans [16]. Thus, in our study, we initially analyze the expression pattern of CMS4 markers in patient-derived organoids (PDO), which represents a modern method to study human cancers. Of these markers, we then focused on the characterization of HTR2B + cells when changing microenvironmental niche factors. Interestingly, we observed a dual role of serotonin and HTR2B stimulation under different conditions, and we found shared features of HTR2B + cells with tumor cells expressing NOTCH3. Thus, our results describe the complex role of serotonin in CRC development, depending on the local microenvironment.

## Materials and methods

### Cell cultures

Normal human colon fibroblasts (NCF) (CCD-18Co, CRL-1459, ATCC, Manassas, VA, USA) were cultured in DMEM, 4500 g/L glucose, GlutaMAX (Gibco, Thermo Fisher Scientific, Waltham, MA, USA), 10% FBS (Biosera, Kansas, MO, USA), 1% penicillin/streptomycin (Gibco) and 1% glutamine (Merck, Darmstadt, Germany). Cells were washed with phosphate-buffered saline (PBS) three times and cultured in a serum-free medium for ELISA (see below). The cell number was counted in a Burker chamber. We only used cells with low (< p12) passage numbers. Cell cultures used in our studies were negative for Mycoplasma contamination tested with Hoechst staining. In some experiments, patient-derived CAFs were cultured for RNA isolation. CAF isolation, characterization, and patients’ data have been published (patient #1,2,4,6,7,8 from [17], Table S1).

### Human CRC organoid cultures and quantification of organoid size

The Medical Research Council of Hungary (ETT-TUKEB, No 51323-4/2015/EKU) approved the experiments with human samples, and informed consent was obtained from patients. We used the CRC organoid lines published by our research group (see characterization of the organoids and patient data in [5, 18] and Table S1). Organoids were cultured in ‘CRC medium’ composed of advanced DMEM/F12 (Thermo Fisher Scientific, Gibco), 10 mM HEPES (Merck), glutamine, penicillin/streptomycin, B27 supplement (Gibco), 10 mM nicotinamide (Merck), 1 mM N-Acetyl-Cysteine (Merck), 50 ng/mL EGF (Peprotech), 10 µM SB202190-monohydrochloride (Merck), and 500 nM A83-01 (Merck). For anoikis prevention, the Rho kinase inhibitor Y27632 (Merck) was added for 3 days after passaging the organoids. In certain experiments, after 6 days of growing in the CRC medium, organoids had their medium changed to a glucose-free or amino acid-free medium. Glucose-free medium was composed of DMEM without glucose and pyruvate (Gibco), 10mM HEPES (Merck), glutamine, penicillin/streptomycin, 10mM nicotinamide (Merck), and 1mM N-Acetyl-Cysteine (Merck). Amino acid-free medium was composed of EBSS-2888, 10mM HEPES, penicillin/streptomycin, 10mM nicotinamide, and 1mM N-Acetyl-Cysteine (Merck). Organoids were removed from the 3D matrix (Matrigel, Corning, New York, NY, USA) every 5 to 7 days mechanically; they were centrifuged at 600× *g* for 5 min, washed with PBS, and digested with TrypLE (Thermo Fisher) until organoids were dissociated into cell clusters with 5–10 cells. Samples were then washed and embedded into the 3D matrix again. When indicated, CRC organoid cells and CCD-18Co NCFs (10,000:20,000 cells, 1:2 ratio) were mixed before embedding them into Matrigel as co-cultures in ’CRC medium’. In some experiments, organoid cultures were treated with an HTR2B inhibitor (10 µM, RS-127445, Selleckchem, Houston, TX, USA or SB-204741, MedChemExpress, Monmouth Junction, NJ, USA), serotonin (10 µM, Merck), HTR2B agonist (10µM, α-methylserotonin maleate, Merck), 5-fluorouracil (5-FU, 1 or 10 µM, MedChemExpress) or Notch inhibitors (γ-secretase inhibitors, DAPT and DBZ, 10 µM, MedChemExpress).

To establish normal colon organoid cultures, samples were collected from patients undergoing CRC surgical operation at a distance of > 3 cm from the tumors. Human colonic crypts were isolated according to [19], and they were embedded into Matrigel droplets (20 µL/well, 48-well plate, Eppendorf, Austria). Organoids were cultured in CRC medium supplemented with 100 ng/mL human noggin (Peprotech, Thermo Fisher), 1000 ng/mL human R-Spondin1 (R&D Systems, Bio-Techne, Minneapolis, Minnesota, United States), 100 ng/mL murine Wnt3a (Peprotech), and 1 nM gastrin (Merck). Organoid cultures were split weekly by mechanical pipetting.

CRC organoids were imaged at 10 times magnification, and the area and diameter of cell clusters and their invasions, along with a count of organoids per image, were measured with the ImageJ software (National Institutes of Health, USA). Briefly, we used the freehand selections tool to manually cover the organoid first, and then the organoid with its invasion area. With each step, the ImageJ software created a convex hull using the ‘gift wrapping algorithm’. A convex hull refers to the simplest convex polygon connecting all of the points in the set. Out of the produced data, we then used the area parameter, which represents the number of square pixels within the convex hull. For each organoid line and treatment, 20–25 organoids were analyzed when measuring organoid and invasion areas.

### Collagen-Based organoid cultures

To prepare 100 µL collagen I matrix, 60 µL distilled water, 10 µL 10× MEM (Gibco), and 30 µL collagen type I (Ibidi, Gräfelfing, Germany) were mixed, and the pH was set to 7.2 with 1 M NaOH. Single cells or mature organoids isolated from Matrigel, or collagen-I matrix, were embedded in this collagen mixture without mechanical disruption. For producing floating collagen cultures, we used a previously published protocol [20].

### Flow cytometry and cell sorting

Organoids grown in collagen were isolated with 800 µg/ml collagenase II (Merck), then dissociated into single cells by TrypLE. No collagenase was used when organoids were cultured in Matrigel. When analyzing tumor tissue, samples were dissociated with the Tumor Dissociation Kit (Miltenyi Biotech, Bergisch Gladbach, Germany) according to the manufacturer’s protocol. Cells were then suspended in FACS buffer (PBS with 1 mM EDTA, 25 mM HEPES, 1% bovine serum albumin (BSA)) and labeled with antibodies for 20 min. For detecting intracellular antigens with flow cytometry, cells were fixed in 4% paraformaldehyde (PFA) in PBS for 20 min, permeabilized with 0.1% saponin in PBS, and then labeled with antibodies in 0.1% saponin. 5,000–10,000 events were measured with a Cytoflex (Beckman Coulter, Brea, CA, USA) instrument, or cell subpopulations were sorted by a Sony SH800S cell sorter (Sony Biotechnology, Bothell, WA, USA) in Qiazol lysis buffer (Qiagen, Hilden, Germany) for RNA isolation or in DMEM with 10% FBS, 1% penicillin/streptomycin, 25 mM HEPES and glutamine for further culturing. Sorted cells were centrifuged at 300 g for 10 min at 4 °C and embedded in 20 µL Matrigel or collagen at 8,000–15,000 cells/well with identical cell numbers for each condition in the same experiment. Antibodies are listed in Table S2.

### Whole-Mount immunostaining

Organoids were cultured in 8-well chamber slides (BD Biosciences, East Rutherford, NJ, USA), fixed in 4% PFA for 40 min, washed with PBS, and blocked and permeabilized with whole-mount blocking buffer (WBB: 5% FBS, 0.2% BSA, 0.3% Triton X-100, in PBS) for 30 min. Samples were incubated with primary antibodies at 4 °C overnight in WBB. After washing, samples were incubated with secondary antibodies in WBB for 2 h at room temperature (RT). Organoids were then mounted with ProLong Diamond antifade mountant containing DAPI (Thermo Fisher Scientific). Confocal images were taken with a Leica TCS SP8 microscope, and we used the ImageJ software for analysis and quantification. We analyzed 5–6 images and 2–5 organoids/image for each PDO line and treatment condition. Antibodies are listed in Table S2.

### Immunofluorescent staining of paraffin-embedded sections

Based on standard protocols, PFA-fixed and paraffin-embedded sections were deparaffinized and rehydrated. Sections were then boiled in Tris-EDTA high-pH buffer (10 mM Tris base, 1 mM EDTA solution, 0.05% Tween-20 in distilled water, pH = 9.0) for 15 min and allowed to cool to RT for 20 min. After washing, sections were blocked in Tris-buffered saline containing 0.1% Tween-20 (TBS-T), primary antibodies were applied at 4 °C overnight, and secondary antibodies were applied for 2 h at room temperature (all in TBS-T). Samples were covered with ProLong Diamond antifade mountant containing DAPI (Thermo Fisher Scientific).

### ELISA

Conditioned medium from PDOs or fibroblasts (cultured serum-free) was collected after 48 h, cell debris was removed by centrifugation at 300 g for 5 min, and serotonin detection was carried out according to the manufacturer’s protocol (serotonin ELISA kit, Abnova, Taoyuan City, Taiwan). Matrigel droplets without organoids were used as controls. We measured the OD values on a HiPo MPP-96 Microplate Photometer (Biosan, Riga, Latvia).

### RNA isolation and expression analysis

Total RNA was isolated with the miRNEasy Micro Kit (Qiagen) following the manufacturer’s instructions in 15 µL RNAse-free water. Cells were directly sorted into Qiazol lysis buffer (Qiagen) in some experiments. The RNA concentration was measured with a NanoDrop instrument (Thermo Fisher). We prepared cDNA with the Sensi-FAST cDNA Synthesis Kit (Bioline, Meridian Bioscience, London, UK) from 300 ng RNA (in 20 µL final volume). The SensiFAST Sybr No-Rox Kit (Bioline) was used to carry out quantitative PCR reactions via a BioRad (Hercules, CA, USA) CFX384 Touch real-time PCR instrument (384-well format, 5 µL/well volume) using the SybrGreen method with the annealing at 60 °C. Results were evaluated according to this equation: relative expression level = 2^−ΔCt^, where ΔCt = Ct(gene of interest)—Ct(housekeeping gene). The primers are listed in Table S3.

### Viability assays

5,000 organoid cells were embedded in 6 µL 3D Matrigel or collagen-I in 96-well plates, cultured for 3 days in CRC medium, and then changed to CRC medium, glucose-free, or amino acid-free medium with treatments. Serotonin, HTR2B agonist (α-methylserotonin), HTR2B inhibitor 1 (RS-127445), HTR2B inhibitor 2 (SB-204741), and Notch inhibitors (γ-secretase inhibitors, DAPT and DBZ, 10 µM, MedChemExpress) were the compounds of interest. In some experiments, the inhibitor was given for 1 h before adding serotonin or the agonist. After 3 days, organoid culture viability was determined with the CellTiterGlo 3D Cell Viability Assay (Promega, Madison, WI, USA) according to the manufacturer’s protocol, and we scanned the plate with a Fluoroskan FL (Thermo Fisher Scientific) instrument. Viability results were evaluated with the following equation: well viability % = (well value—average positive control)/(average vehicle control—average positive control) × 100. DMSO served as the vehicle control, 5 µM staurosporine (MedChemExpress) as the positive control [17]. Samples were run in four technical replicates and they were averaged. Alternatively, in some experiments, cells dissociated from PDOs were incubated with LIVE/DEAD™ Fixable Blue Dead Cell Stain Kit (Thermo Fisher) for 30 min according to the manufacturer’s protocol and were analyzed with flow cytometry.

### Bioinformatical and statistical analysis

The GSE17537 and GSE14333 data sets (https://www.ncbi.nlm.nih.gov/geo) were used for survival analysis. They were unified, and expression levels were z-score transformed before carrying out Kaplan–Meier analysis and log-rank test with SPSS 29.0.1.0 statistical software (IBM, Armonk, New York, USA). When comparing CMS groups, we used the GSE39582 set. The Protein Atlas database (www.proteinatlas.org) was analyzed using the ‘quantity’ parameter for immunostaining positivity of tumor cells in CRC tissue sections. For statistical evaluation of our experimental data, Student’s paired or unpaired *t*-tests or ANOVA with Tukey post hoc tests were applied with *p < 0.05, **p < 0.01, and ***p < 0.005 significance levels. Microsoft Excel (Redmond, WA, USA), IBM SPSS version 29 (IBM), and GraphPad software (Boston, MA, USA) were used for statistical evaluation. Mean + SD or median, 25th, and 75th percentiles for the box plots are shown. Unless otherwise stated, data are presented from three-four PDO lines where each dot illustrates one organoid line in all figures (*n* = 3–4, in case of *n* = 3, data were obtained from PDO lines #1–3). When quantifying whole-mount immunostaining, 5–6 images with 2–5 organoids/image were analyzed and summarized as one PDO line for each condition.

## Results

### Intra-tumoral heterogeneity for the CMS4 marker HTR2B in CRC

Several tumor markers characterizing the CMS4 subgroup have recently been described [8, 9]. To test their expression in CRC organoids, we first focused on a classifier containing PDGFRA, PDGFRB, PDGFC, and KIT [8]. As expected, analyzing public datasets that contain not only gene expression data but also CMS classification of patients indicated the higher RNA level KIT, PDGFRB, and PDGFC, but not PDGFRA for CMS4 compared to other CMS groups and normal colon samples (Fig S1A). We detected the RNA of all these markers in PDOs, although at a low level, and they were positive for PDGFRB and PDGFRA at the protein level, too (Fig S1B). In addition, the expression level of any of these markers did not indicate a difference in patient survival (Fig S1C). In contrast, ZEB1, FRMD6, and HTR2B that have been suggested as CMS4 markers by another study [9] predicted patient survival, and high levels of the CMS2/3 marker CDX2 correlated with a better disease outcome (Fig S2A). As expected, whereas we observed a higher expression of CDX2 in CMS2/3 patients, ZEB1, FRMD6, and HTR2B displayed an elevated level in CMS4 samples in public datasets (Fig S2B). The analysis of the Protein Atlas database (www.proteinatlas.org) concluded that FRMD6 showed no intra-tumoral heterogeneity, and all tissue sections were uniformly positive for this molecule. Furthermore, ZEB1 had ambiguous results, with some antibodies detecting heterogeneous expression and others detecting a complete absence of expression in the tumor cells. In contrast, tumor cells were heterogeneously positive for CDX2 and HTR2B immunostaining (Fig S2C).

**Fig. 1:**
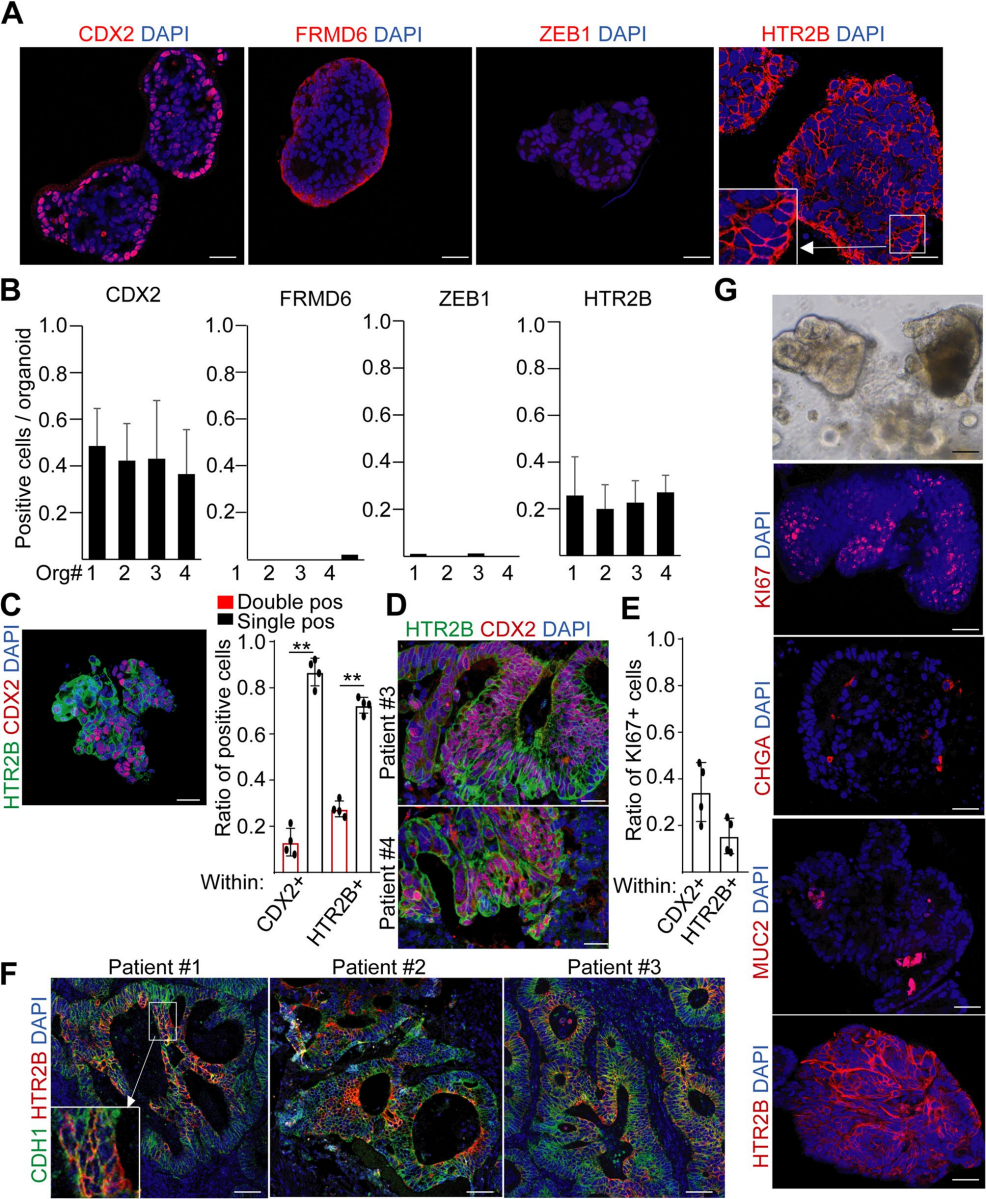
The CMS4 marker HTR2B shows an intra-tumoral cellular heterogeneity in CRC. **A**-**B** Representative confocal microscopic images (**A**) and their quantification (**B**) in PDO lines for CDX2, FRMD6, ZEB1, and HTR2B. **C** The ratio of single and double positive organoid cells for CDX2 and HTR2B within the CDX2 + or HTR2B + populations (representative confocal image and quantification). **D** Immunostaining for CDX2 and HTR2B from CRC tissue slides. **E** The ratio of KI67 + proliferating cells within the CDX2 + or HTR2B + PDO cells (analysis of confocal images). **F** Immunohistochemistry of tissue slides from three CRC patients for the indicated markers. CDH1 was used as an epithelial cell-specific positive control. Note the epithelial cell restricted heterogeneous expression of HTR2B. **G** Light microscopic and confocal images from normal human colonic organoids. MUC2 and CHGA are markers of differentiated Goblet and enteroendocrine cells, respectively. Student t-test (**C**) was carried out with **p < 0.01. Scale bars: 20 μm (**A**, **C**, **G**) or 100 μm (**D**, **F**). *n* = 4 (**C**, **E**). For **B**), 10–15 organoids were evaluated from each PDO line

While in our patient-derived organoids neither ZEB1 nor FRMD6 were detected at the protein level, HTR2B and CDX2 were heterogeneously expressed and present in all organoid lines (Fig. 1A-B). Furthermore, only a subpopulation of tumor cells was positive for both markers in PDOs (Fig. 1C), and we observed only a partial overlap between these two molecules in CRC tissue sections as well (Fig. 1D). In addition, CDX2 + and HTR2B + cells only partially overlapped with the proliferating KI67 + CRC population (Fig. 1E).

Next, we focused on the expression pattern of the CMS4 marker HTR2B. We confirmed the presence of this molecule in CRC organoids with three different antibodies recognizing either the intracellular and extracellular domains of the protein (Fig. S2D). Immunohistochemical analysis of tissue slides confirmed that HTR2B was absent in the mesenchymal stromal cells but present at a varying level on CRC tumor cells, and it displayed a plasma membrane-bound staining pattern (Fig. 1F). In addition, organoids established from normal colon also contained HTR2B + epithelial cells (Fig. 1G), suggesting that HTR2B is not specific for CRC. Of note, these organoids contained mucin-2+ (MUC2+) and chromogranin A+ (CHGA+) differentiated cells, as well as proliferating cells in the crypt-like structures, proving the viability of our normal colon organoids (Fig. 1G). Thus, we concluded that CDX2 and HTR2B, markers of the CMS2/3 and CMS4 subgroups, respectively, are present in the same tumor samples.

### Neither CRC cells nor fibroblasts are a major source of serotonin

PDAC tumor cells express genes for serotonin production and uptake at a high level, and they are an important source of serotonin in unfavorable conditions [15]. However, we found that neither fibroblasts nor CRC cells produced significant quantities of serotonin in normal or unfavorable conditions, such as glucose-free or amino acid-free medium (data not shown). We observed the reduced expression of TPH1, the rate-limiting enzyme of serotonin synthesis, and SLC18A2, which relates to serotonin cellular secretion, in CRC PDOs compared to normal colon organoids (Fig S3A). CRC organoids had a higher expression of SLC6A4, involved in serotonin re-uptake, but showed no difference in the RNA level of the serotonin-degrading MAOA enzyme (Fig S3A). This implies that CRC organoid cells may be able to metabolize serotonin.

Neuroendocrine cells are a major source of serotonin in normal intestine, and recent studies indicated that CRC progresses through a high plasticity cell state which enables the expression of non-canonical genetic programs, such as neuroendocrine cell-specific genes in the tumors [21]. To test whether the CMS4 CRC subtype may accumulate tumor cells differentiated into neuroendocrine cells, we used this neuroendocrine genetic program (Table S4), however, we found no overlap with genes characterizing the CMS4 subgroup [22]. We did not detect an overlap between the neuroendocrine program and CMS4 genes specific only for tumor cells either (gene list from [2], Table S4, Fig S3B). Thus, in contrast to PDAC, neither CRC cells nor fibroblasts represent a major source of serotonin, and this molecule may derive from the surrounding normal colon tissue or other organs, highlighting the large variance among different tumor types. Although previous reports applied inhibitors of HTR2B [13, 14] in cell cultures, based on our results, we focused on activating this receptor in our PDO model system in our subsequent experiments.

### Activating HTR2B reduces tumor cell survival under unfavorable conditions

The activation of the mTOR pathway and the consequently higher phosphorylation of the mTORC1 target ribosomal protein S6 (P-S6) are critical in cell survival. Interestingly, the mTORC1 inhibitor rapamycin had no dramatic effect on PDO forming efficiency and organoid size when starting either from single cells or from small cell clusters, independently of whether rapamycin was added immediately or only 3 days after starting the cultures (Fig S4A). This suggests that the mTORC1 pathway is not critical for PDO colony formation and CRC cell survival under optimal conditions. We proved the effect of rapamycin by detecting a reduced percentage of P-S6 + cells by microscopy (Fig S4B), and we also obtained similar results by flow cytometry, which provides an easy method for detecting P-S6 + cells. These experiments also highlighted the significant heterogeneity in the ratio of P-S6 + cells among PDO lines (Fig S4C).

**Fig. 2:**
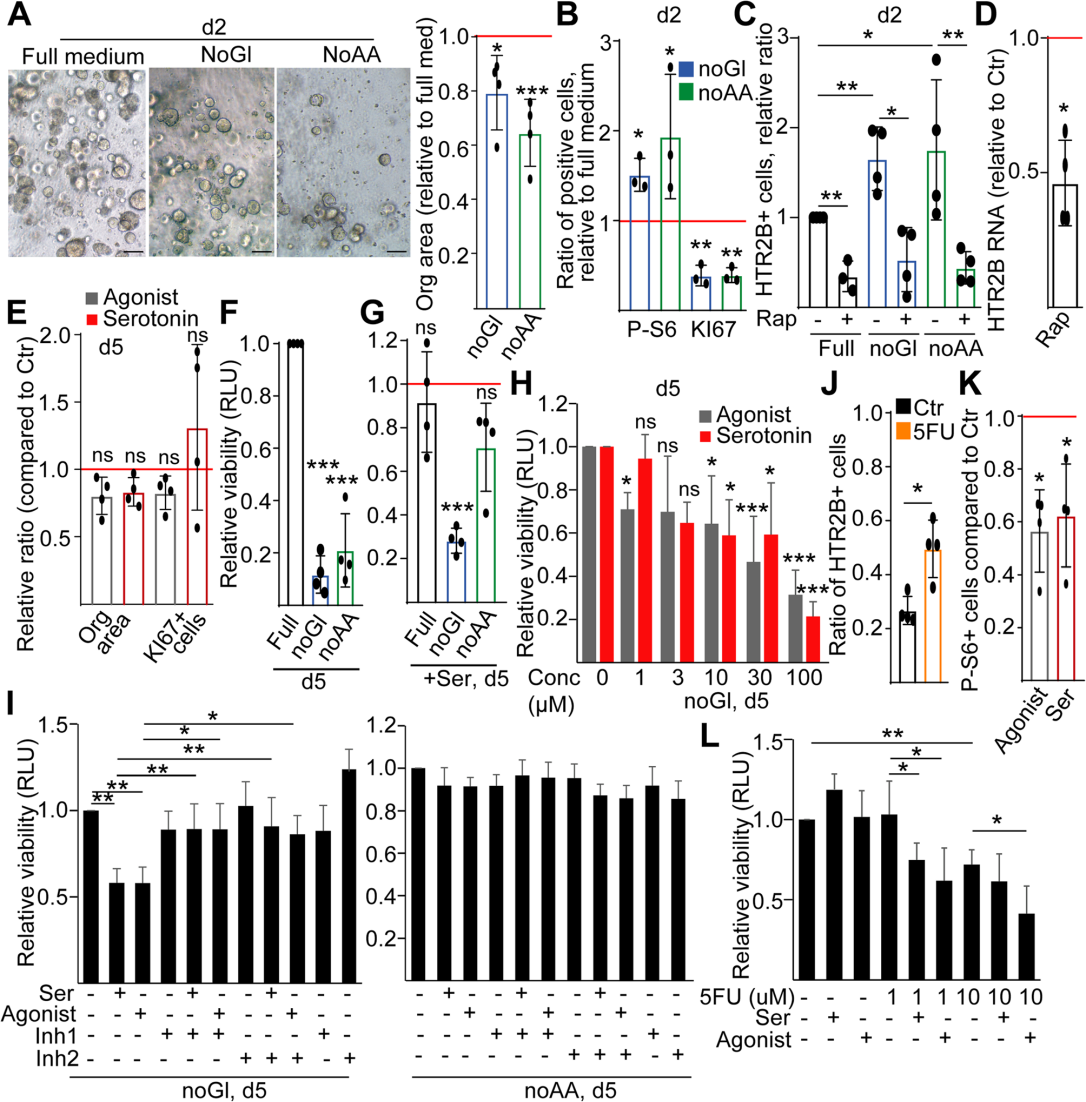
Unfavorable conditions increase the percentage of HTR2B + cells and lead to a reduced viability of tumor cells induced by serotonin. **A** Light microscopy images of PDO cultures in control (full), in carbohydrate-free (noGl), and in amino acid-free medium (noAA) and the quantification of relative organoid area compared to the full medium control. The horizontal red line indicates the control with a relative area of 1. **B** The relative proportion of KI67 + or P-S6 + cells within organoid lines cultured in noGl or noAA medium, compared to full control (horizontal red line). Analysis of confocal images. **C** The effect of the mTOR inhibitor rapamycin on the ratio of HTR2B + cells under the indicated conditions. For each organoid line, samples were compared to the untreated full medium control (flow cytometry). **D** Relative RNA level of HTR2B in the presence of rapamycin (1µM, 2 days, RT-qPCR). Values were normalized to the housekeeping gene, and they were then compared to the untreated control (red line). **E** Changes in the organoid area and the ratio of KI67 + proliferating cells in PDO lines when cultured in full medium for 5 days (analyzed from light and confocal microscopy images). **F** The effect of different culturing conditions on cell viability, measured by CellTiterGlo 3D. **G** The effect of serotonin (10µM) on PDO viability compared to untreated samples under different culturing conditions. **H** Concentration dependent effect of serotonin and agonist, detected by CellTiterGlo 3D test. **I** Cell viability in the presence/absence of serotonin (10µM), α-methylserotonin (agonist, 10µM), and HTR2B inhibitors (inhibitor 1: RS-1274451, inhibitor 2: SB 204741, both at 1µM) in carbohydrate (noGl) or amino acid-free medium (noAA). **J** Ratio of HTR2B + cells in the presence or absence of 5-FU. Note that 5-FU was applied at an IC50 concentration for each PDO line that had been determined in our previous studies ([17] and Table S5). Samples were processed after 3 days of treatment (flow cytometry). **K** Relative changes in the ratio of P-S6 + cells in the presence of serotonin (10µM) or HTR2B agonist (α-methylserotonin, 10µM). The horizontal line indicates the control (flow cytometry). **L** Relative cell viability in the presence of the indicated treatments, compared to control samples (CellTiterGlo 3D analysis). Scale bars: 50 μm. Paired (**A**-**G** and **K**), unpaired t-test (**J**) or ANOVA with Tukey post hoc test (**H**, **I**, **L**) were applied with *p < 0.05, **p < 0.01, ***p < 0.005, ns: *p* > 0.05. *n* = 4 (exception: *n* = 3 for B)

HTR2B plays a critical role in the survival of PDAC cells under unfavorable conditions [15]. Thus, we explored the potential expansion of the HTR2B + cell population under glucose or amino acid-deprived conditions. Since only very few organoids were formed from single-cell suspensions in unfavorable conditions, we set up cultures from already established organoids in glucose or amino acid-free medium. A previous study proved that dying cells induced mTOR activation in the neighboring cells by a paracrine mechanism, providing a key step in CRC tumor cell survival and resistance to drugs [23]. We detected a reduction in organoid size and the ratio of KI67 + cells already after 2 days in glucose or amino acid-free medium, which was paralleled by an increased percentage of P-S6 + cells within the surviving cell population (Fig. 2A-B), suggesting that in contrast to standard culturing medium, mTORC1 may be important under unfavorable conditions. We also observed a higher number of HTR2B + cells in the absence of glucose or amino acid, and the size of the HTR2B + population was reduced in all conditions when applying rapamycin (Fig. 2C). Rapamycin also decreased the RNA level of HTR2B (Fig. 2D), indicating that HTR2B is under the direct control of the mTORC1 pathway. Importantly, we also confirmed the reduction of the ratio of P-S6 + and HTR2B + cells when applying everolimus which is another clinically used mTORC1 inhibitor (Fig. S4D).

To test the effect of HTR2B stimulation, we next applied serotonin or the agonist of this receptor. Surprisingly, no significant change was observed on organoid size and cell proliferation in optimal conditions (Fig. 2E). However, cell survival dramatically decreased 5 days after culturing in glucose or amino acid-free medium (Fig. 2F), and serotonin further reduced survival only in the absence of glucose but not in complete medium (Fig. 2G). The effect of both serotonin and the agonist was dose-dependent, and they could be antagonized by applying two different HTR2B inhibitors in glucose-free but not in amino acid-free conditions (Fig. 2H-I). Interestingly, the percentage of HTR2B + cells also increased when applying the clinically used 5-fluorouracil (5-FU) at a previously determined concentration where only a fraction of tumor cells undergo apoptosis (IC50 dose [17],, Fig. 2J and Table S5). Although activating HTR2B did not affect organoid size and cell proliferation (see Fig. 2E), it reduced the ratio of P-S6 + cells in normal conditions (Fig. 2K). In line with these data, serotonin or the HTR2B agonist decreased cell survival even at a 5-FU concentration where this drug alone had no dramatic effect yet (Fig. 2L). Collectively, our data indicate that HTR2B expression is induced in unfavorable conditions, and stimulating HTR2B has an inhibitory effect on cell survival in some specific conditions, such as in the absence of glucose or the presence of 5-FU.

### Fibroblasts and collagen deposition critically modify the percentage of HTR2B + cells, and HTR2B induces invasion in collagen

**Fig. 3:**
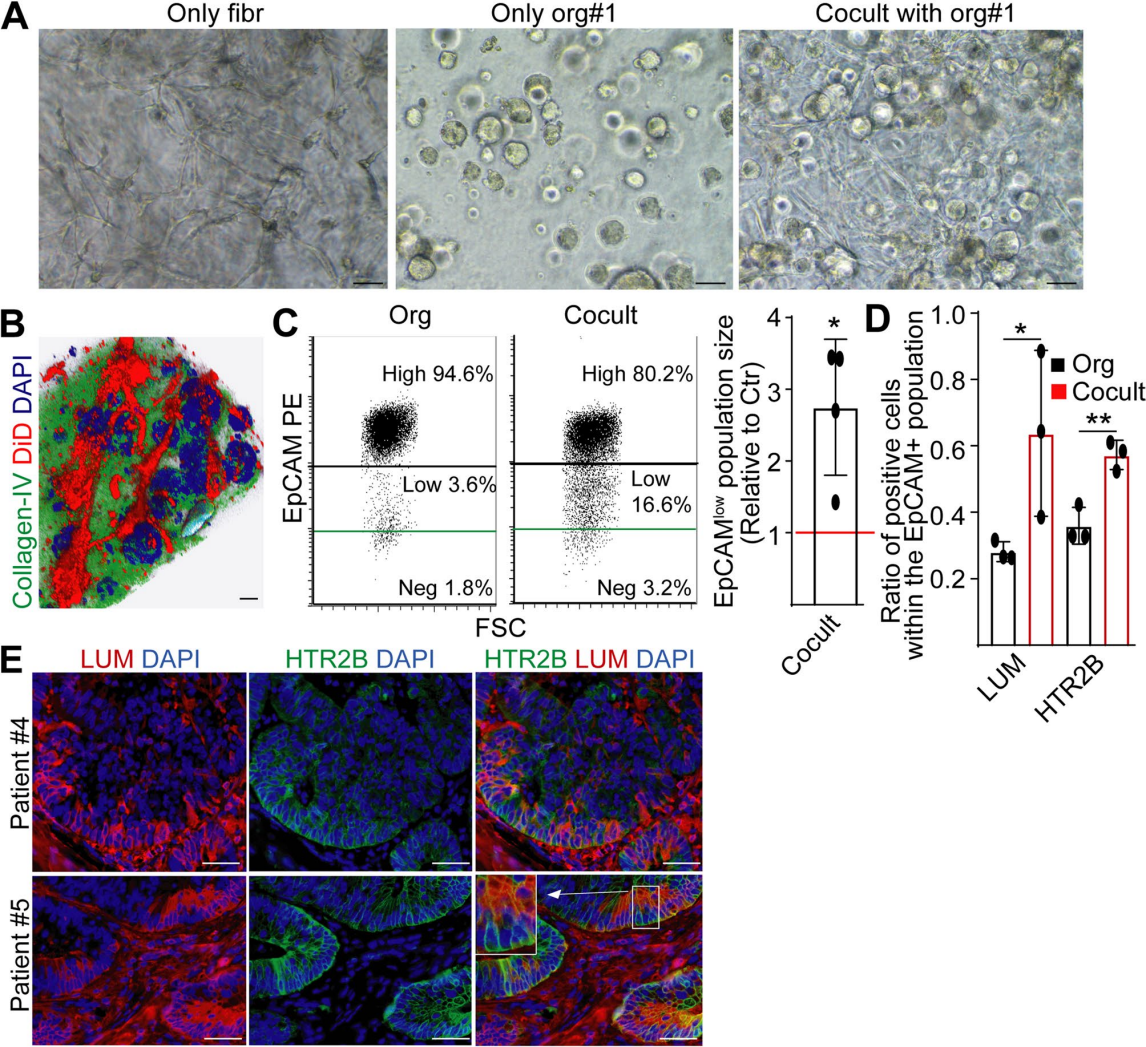
Fibroblasts induce a partial EMT and an increased HTR2B expression in CRC cells. **A** Light microscopy images from 3D cultures of fibroblasts, PDOs, or their co-cultures. **B** Confocal microscopic image of a co-culture. Fibroblasts were labeled with the membrane staining dye DiD before co-culturing (3D reconstruction, background immunostaining for collagen-IV). Note the close proximity of fibroblasts and organoids. **C** Analysis of cell surface EpCAM level from cultures containing only organoids or co-cultures with fibroblasts (representative flow cytometry dot plots and their quantification). Note that EpCAM is an epithelial cell marker. Isotype control samples were used to determine the unspecific background (green line). **D** Relative ratio of LUM or HTR2B positive cells in PDOs and co-cultures when gating for the EpCAM + cells in flow cytometry. Note that fibroblasts do not express the epithelial marker EpCAM. **E** Immunostaining of CRC tissue sections for the indicated markers. Scale bars: 50 μm (**A**), 20 μm (**B**), 25 μm (**E**). Paired (**C**) or unpaired (**D**) t-tests were used with *p < 0.05, **p < 0.01. *n* = 3 (**D**) or *n* = 4 (**C**)

The CMS4 subtype and poor patient survival prognosis are closely associated with EMT and fibroblast accumulation. To test the effect of fibroblasts on the size of the HTR2B + cell population, we next prepared co-cultures with organoids (Fig. 3A). Fibroblasts marked with a membrane labeling dye proved the close proximity and, in some cases, the direct contact of the two cell types in these co-cultures (Fig. 3B). These co-cultures contained a higher number of cells with only a low cell surface level of the epithelial molecule EpCAM while having an increased percentage of CRC cells with positivity for lumican (LUM) that had been proven as an EMT marker in CRC [24, 25] (Fig. 3C-D), indicating the partial EMT (pEMT) in these cultures. Notably, co-cultures increased the size of the HTR2B + CRC cell population within the EpCAM+ (EpCAM^low^ and EpCAM^high^) tumor cells, too (Fig. 3D). We also observed LUM+/HTR2B + tumor cells close to the mesenchymal component in CRC tissues (Fig. 3E). Whereas conditioned medium from fibroblasts affected the ratio of cells positive for LUM, we detected no difference in the percentage of HTR2B + cells, and, as expected, the proportion of P-S6 + cells did not change either (Fig S4E). Thus, soluble molecules secreted by fibroblasts are not the major factors inducing HTR2B expression.

**Fig. 4:**
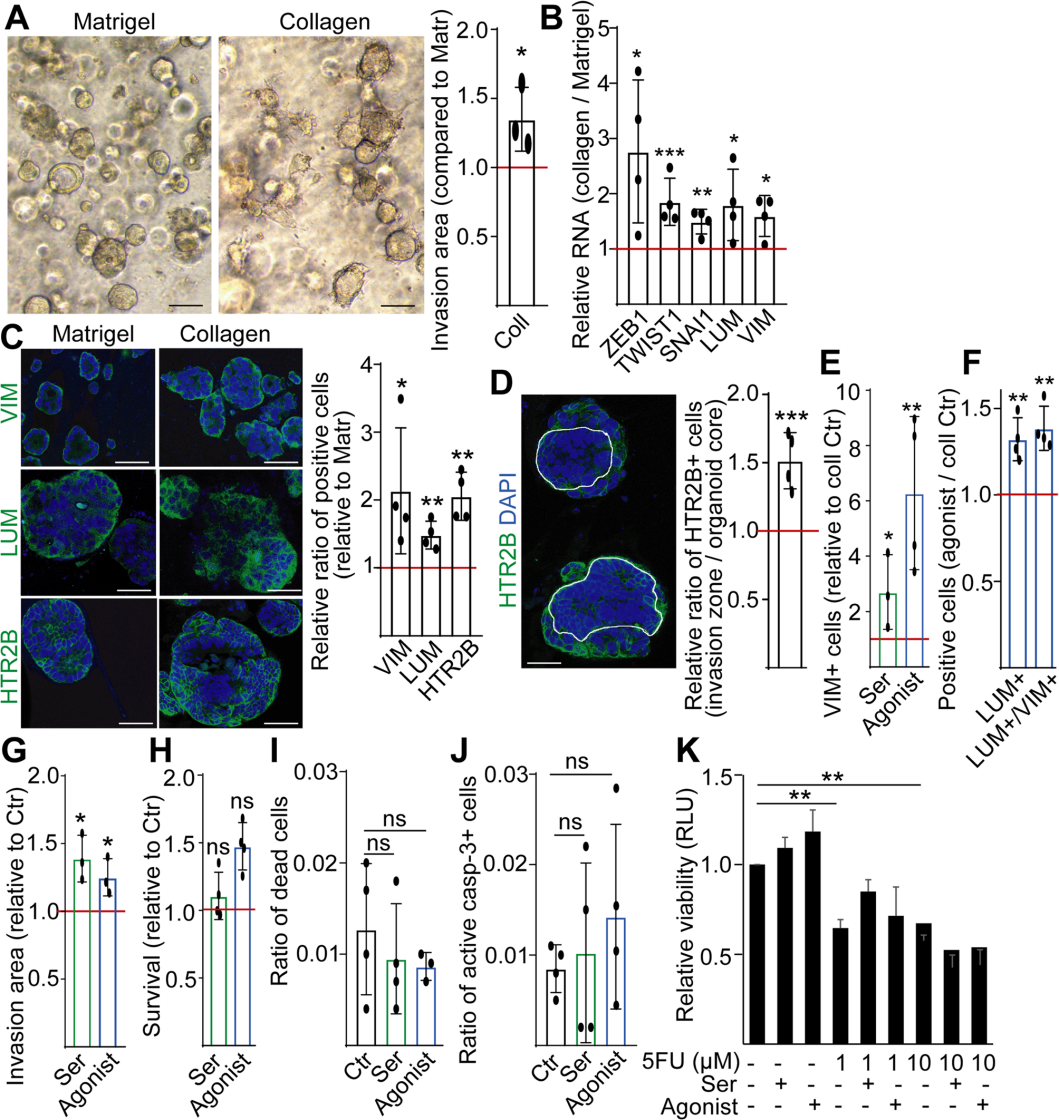
Activating HTR2B induces invasion of PDO cells in collagen-I. **A** Morphology of PDOs in Matrigel and collagen-I (left panel) and the relative invasion area in collagen-I (right panel). For comparison, the horizontal line indicates Matrigel control. For measuring invasion area, see Materials and Methods. **B** RNA level of the indicated genes in PDOs cultured in collagen, compared to Matrigel (red line) (RT-qPCR). **C** Relative ratio of VIM+, LUM + or HTR2B + cells when cultured in collagen-I, compared to Matrigel (representative confocal microscope images and their quantification). **D** Ratio of HTR2B + cells in the invasion zone compared to the organoid core (confocal microscopy, collagen-I cultures, representative image, and statistical evaluation). The yellow line shows the segmentation between the organoid core and the invasion zone. **E** The effect of serotonin or agonist (10µM) on the proportion of VIM + cells (analysis of confocal microscopy images). **F** The effect of HTR2B agonist on the ratio of LUM + and LUM+/VIM + double positive cells in the organoid lines. **G** Changes in the invasion area in collagen-I. **H** The effect of serotonin and HTR2B agonist compared to control samples in collagen, measured by CellTiterGlo 3D. **I** The ratio of dead cells in the presence of the indicated treatments in collagen (cell viability test: LIVE/DEAD™ Fixable Blue Dead Cell Stain Kit and flow cytometry). **J** The proportion of the apoptotic active caspase-3 + cells in collagen (flow cytometry). **K** Viability in the presence of the indicated treatments in collagen-I (CellTiterGlo 3D assay). Scale bars: 50 μm (**A**), 25 μm (**C**-**D**). Paired (**A**-**H**), unpaired t-tests (**I**-**J**) or ANOVA with Tukey post hoc test (**K**) were applied with *p < 0.05, **p < 0.01, ***p < 0.005. *n* = 4 (*n* = 3 for G)

Matrigel, enriched for collagen-IV and laminin, typically does not provide an invasive environment for CRC organoids. Fibroblasts are a rich source of not only soluble molecules, but also ECM components, such as collagen-I. Previously, we provided evidence that CRC cells acquire pEMT in collagen-I with a partially invasive phenotype and an invasion area around the organoids ([17] and Fig. 4A). In line with these results, we observed increased RNA for several EMT genes (Fig. 4B), and more positive cells for the EMT markers vimentin (VIM) and LUM in collagen (Fig. 4C), confirming pEMT at the protein level, too. Similar to co-cultures with fibroblasts, collagen-I led to a higher proportion of HTR2B + CRC cells (Fig. 4C). We also observed an increased ratio of LUM + and HTR2B + PDO cells in another collagen-based assay applying floating collagen droplets ([20], Fig S4F). Importantly, the invasion zone contained a higher number of HTR2B + cells compared to the organoid core in collagen (Fig. 4D), showing the accumulation of HTR2B + cells within the migratory cells. Treating cultures with serotonin or an HTR2B agonist increased the number of cells with EMT markers and the invasion area in collagen (Fig. 4E-G) without reducing cell survival and without inducing apoptosis (Fig. 4H-J). In line with these data, serotonin or an HTR2B agonist did not further decrease cell survival, which was reduced by 5-FU in collagen (Fig. 4K). In summary, these data indicate that the effects of fibroblasts on the increase of the HTR2B + cell population size may be mediated by collagen-I, and activating HTR2B induces cellular migration in a permissive ECM background.

### HTR2B^high^ and NOTCH3^high^ tumor cells share common features in a permissive microenvironment

**Fig. 5:**
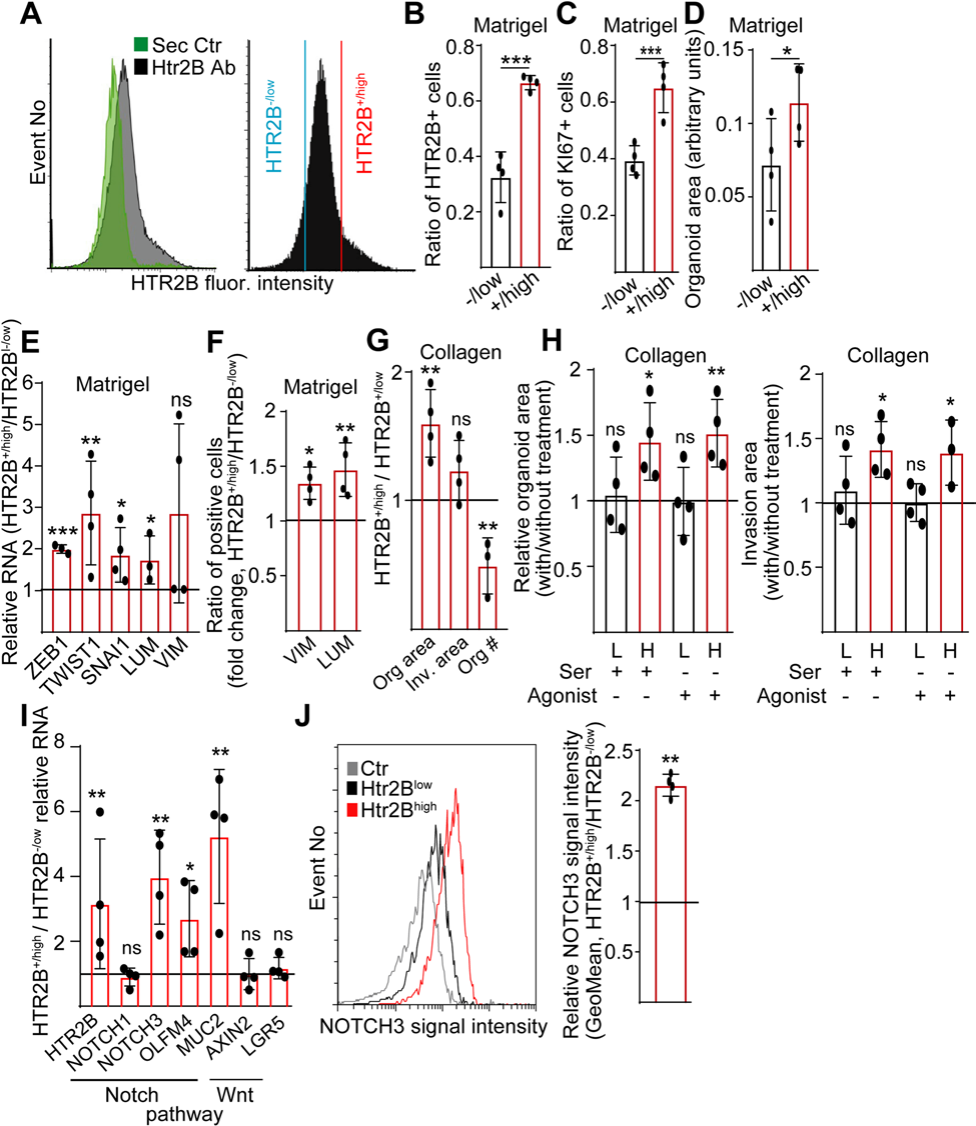
HTR2B^+/high^ tumor cells have a higher invasion potential compared to the HTR2B^-/low^ population. **A** Representative flow cytometry histogram and the sorting strategy. **B**-**C** Ratio of HTR2B+ (**B**) or KI67 + proliferating cells (**C**) in the organoids derived from sorted HTR2B^+/high^ and HTR2B^-/low^ PDO cells, analyzed after 7 days in Matrigel (confocal microscopy). **D** The area of organoids from sorted cells (light microscopic images, analysis with ImageJ). **E** RNA level of the indicated genes in HTR2B^+/high^ cell derived organoids compared to the HTR2B^-/low^ organoids (RT-qPCR). **F** VIM + and LUM + cells in the HTR2B^+/high^ cell-derived organoids compared to HTR2B^-/low^ organoids (marked with a horizontal line). Analysis was carried out after 7 days in culture (confocal microscopy). **G** Changes in the indicated parameters of HTR2B^+/high^ organoids compared to the HTR2B^-/low^ ones (light microscopic image analysis). **H** The effect of serotonin and HTR2B agonist (10 μM) on HTR2B^+/high^ or HTR2B^-/low^ cell-derived organoids (light microscopy, measured after 7 days in collagen-I, compared to the respective untreated control). Organoid area and invasion area were measured. **I** Relative RNA level of the indicated genes in HTR2B^+/high^ organoids compared to HTR2B^-/low^ PDOs (RT-qPCR). Expression values were first normalized to housekeeping, and then the value from HTR2B^-/low^ PDOs was taken as 1 (horizontal line). **J** NOTCH3 signal intensity in the sorted HTR2B^+/high^ cells compared to the HTR2B^-/low^ population (flow cytometry). Unpaired (B-D) or paired (**E**-**J**) t-tests were used with *p < 0.05, **p < 0.01, ***p < 0.005, ns: *p* > 0.05. *n* = 4

To get an insight into the phenotype of the HTR2B + CRC population, we sorted HTR2B^+/high^ and HTR2B^−/low^ cells (Fig. 5A). Even after 7 days of culturing, HTR2B^+/high^ cell-derived organoids contained more HTR2B + cells (Fig. 5B), confirming that the difference in HTR2B level is maintained in organoid cultures. Interestingly, HTR2B^+/high^ PDOs had a larger size with more proliferating cells than organoids initiated by HTR2B^−/low^ cells (Fig. 5C-D). We measured a higher level of several EMT gene RNAs in HTR2B^+/high^ organoids (Fig. 5E), and they also contained more VIM + and LUM + cells, highlighting the elevated pEMT phenotype (Fig. 5F). In addition, HTR2B^+/high^ cells formed larger organoids in collagen-I but with a diminished organoid-initiating efficiency (Fig. 5G). Stimulating HTR2B with either serotonin or an agonist further increased organoid area and elevated the invasion area only in HTR2B^+/high^ but not in HTR2B^−/low^ cell-derived PDOs (Fig. 5H). Thus, HTR2B^+/high^ cells have higher levels of EMT markers, and activating HTR2B results in invasion in a permissive ECM background.

According to previously published data, NOTCH3 activity induces the aggressive CMS4 CRC subtype [6]. Interestingly, HTR2B^+/high^ organoids expressed NOTCH3 and the NOTCH target genes MUC2 and OLFM4 at a higher intensity. In contrast, we observed no difference in the RNA levels of the Wnt targets AXIN2 and LGR5 compared to HTR2B^−/low^ samples (Fig. 5I). The HTR2B^+/high^ cell population also had a higher cell surface NOTCH3 level than HTR2B^-/low^ sorted cells (Fig. 5J**)**, raising the possibility that HTR2B^+/high^ and NOTCH3^high^ cells are overlapping populations.

**Fig. 6:**
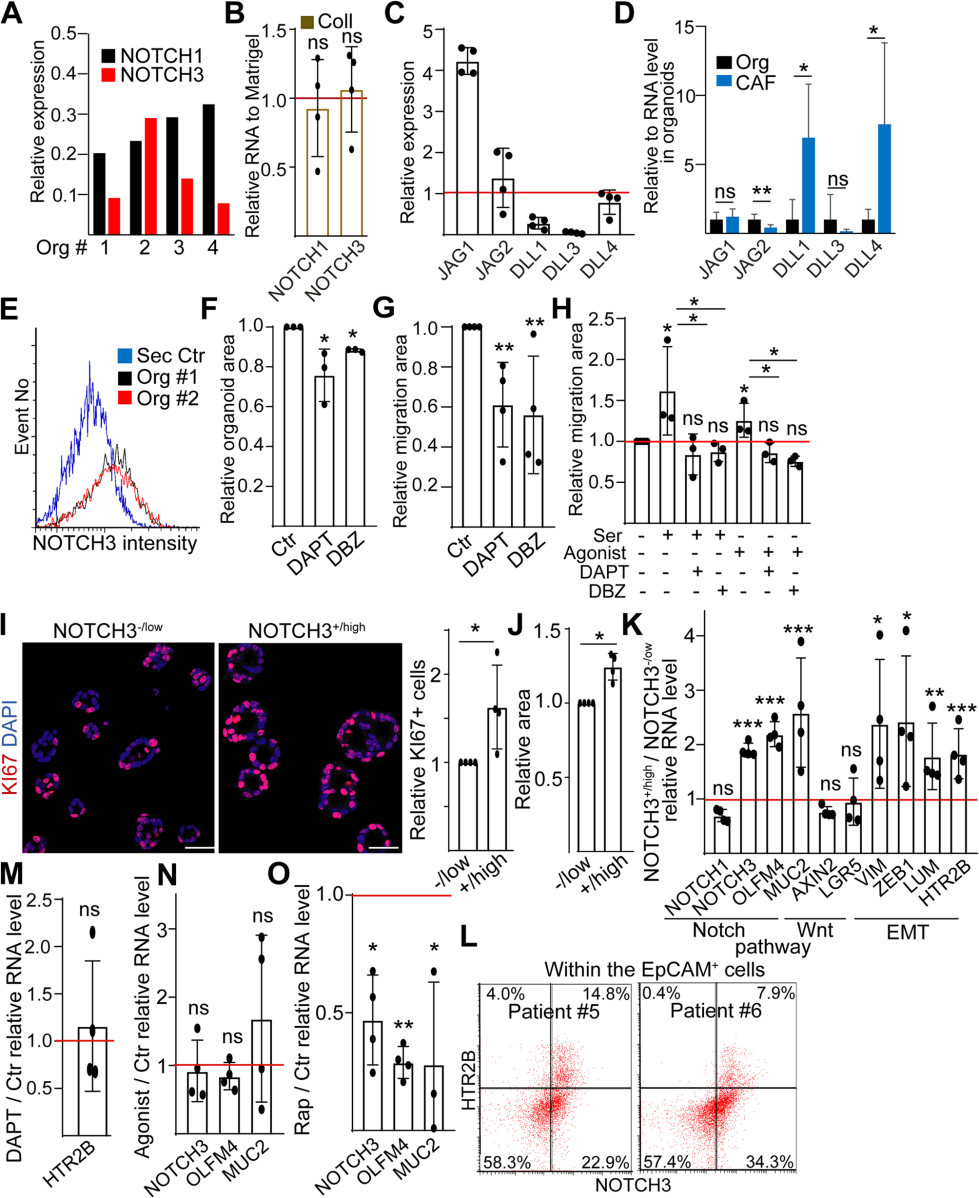
NOTCH3^+/high^ tumor cells share common features with HTR2B^+/high^ cells in a permissive microenvironment. **A** RNA level of NOTCH1 and NOTCH3 in four PDO lines, normalized first to housekeeping and then to AXIN2 (RT-qPCR). Note that the Wnt target gene AXIN2 (used for comparison) is expressed at a high level in CRC cells. **B** RNA level of NOTCH1 and NOTCH3 in collagen compared to Matrigel (RT-qPCR). Data were normalized to housekeeping control, and then Matrigel values were taken as 1. **C** Expression of the indicated genes in PDO lines. Note that data were compared first to housekeeping and then to NOTCH3 to illustrate NOTCH ligand levels relative to their receptor. **D** RNA level of NOTCH ligands in fibroblasts (CAF, *n* = 6) compared to organoids (*n* = 4, RT-qPCR). **E** Flow cytometry histogram of cell surface NOTCH3 level from two different PDO lines. **F**-**G**) The effect of the NOTCH inhibitor DAPT and DBZ (10 µM) on the organoid (**F**) and invasion area (**G**) in collagen-I (light microscopy, image analysis by ImageJ). Note that organoids grown in Matrigel for 4 days were embedded into collagen-I for a further 5 days. **H** Relative invasion area (compared to control) in the presence of the indicated treatments. **I** Representative confocal microscopy images and their quantification for KI67. NOTCH3^+/high^ and NOTCH3^-/low^ cells were sorted, and organoids were analyzed on day 7. **J** Relative area of NOTCH3^+/high^ organoids compared to NOTCH3^-/low^ cell-derived organoids (light microscopy). **K** Fold change in the RNA level of the indicated genes in the NOTCH3^+/high^ compared to NOTCH3^-/low^ organoids (RT-qPCR). Expression data were first normalized to housekeeping, and then values of NOTCH3^-/low^ samples were taken as 1. **L** Flow cytometry from the tumor tissues of two CRC patients. Note that signals for HTR2B and NOTCH3 were analyzed from EpCAM + cells. **M**-**O** Relative RNA of the indicated genes in the presence of DAPT (**M**, 10µM), the HTR2B agonist (**N**, 10µM) or rapamycin (**O**, 1 µM). RT-qPCR data were normalized to housekeeping and then compared to the untreated controls (red line). Scale bars: 20 μm. Unpaired (**D**) or paired (**A**-**C**, **E**-**O**) t-tests were applied with *p < 0.05, **p < 0.01 and ***p < 0.005, ns: *p* > 0.05. *n* = 3–4

To test this hypothesis, we first analyzed immunohistochemical data from the Protein Atlas database (www.proteinatlas.org), and we found that both NOTCH1 and NOTCH3, the two NOTCH receptors that are predominantly expressed in CRC, were heterogeneously present in patient samples (Fig S5A). However, only the level of NOTCH3 correlated negatively with patient survival, and only NOTCH3 expression differed between the CMS4 and other CRC patient groups (Fig S5B-C). PDOs also expressed both NOTCH1 and NOTCH3 receptors at a high level, and their RNA levels did not change when moving organoids from Matrigel into collagen (Fig. 6A-B). We detected NOTCH ligands both in CRC cells and in CAFs, although with a different expression pattern (Fig. 6C-D), suggesting that interactions of CRC cells with other tumor cells or CAFs can activate the NOTCH pathway. Furthermore, we also proved the presence of NOTCH3 on the surface of CRC organoid cells by flow cytometry (Fig. 6E). Interestingly, blocking NOTCH activity with two different inhibitors slightly decreased organoid size, and they reduced the invasion area in collagen (Fig. 6F-G). In addition, NOTCH inhibitors blocked the increase in invasion area induced by serotonin or HTR2B agonist (Fig. 6H). To further characterize tumor cells with NOTCH activity, we produced organoids from sorted NOTCH3^+/high^ and NOTCH3^−/low^ CRC cells. NOTCH3^+/high^ PDOs had more proliferating cells and larger organoid size compared to NOTCH3^−/low^ organoids (Fig. 6I-J). Whereas these PDOs did not differ in AXIN2 and LGR5 RNAs, we detected higher levels of not only the NOTCH targets OLFM4 and MUC2 but also HTR2B and the EMT markers VIM, ZEB1, and LUM (Fig. 6K). In summary, these data provide evidence that HTR2B^+/high^ and NOTCH3^+/high^ CRC cells are overlapping populations with shared common features, and both represent tumor cells with elevated EMT. In line with this notion, we detected NOTCH3+/HTR2B + cells within the EpCAM + epithelial and tumor cell population in CRC tissues (Fig. 6L).

Finally, to study the potential cross-communication between HTR2B and NOTCH activation, we analyzed PDOs cultured in collagen in the presence of an HTR2B agonist or a NOTCH inhibitor. Surprisingly, the agonist had no effect on the expression of NOTCH target genes, and we could not find changes in HTR2B level when blocking NOTCH activity either (Fig. 6M-N). However, similar to HTR2B, inhibiting mTORC1 resulted in a reduced level of NOTCH3 and NOTCH target genes (Fig. 6O). These results suggest that HTR2B and NOTCH3 do not regulate each other directly, but both are, at least partially, under the control of mTORC1.

## Discussion

In this study, we found that CDX2 and HTR2B, markers of the CMS2/3 and CMS4 subgroups, respectively, are present in the same tumor samples. Our data indicate that HTR2B expression is under the control of the mTORC1 pathway, and it is induced in unfavorable conditions, such as the lack of amino acids or carbohydrates or treatment with the clinically used 5-FU. Stimulating HTR2B inhibits cell survival only in some specific conditions, such as in the absence of glucose or in combination with 5-FU treatment. We also observed that the presence of fibroblasts and collagen-I in the ECM increased the size of the HTR2B + cell population, and activating HTR2B induced partial EMT and cellular migration in the permissive collagen ECM background. In addition, HTR2B^+/high^ cells had a higher level of NOTCH3, NOTCH targets, and EMT markers. We also provide evidence that not only HTR2B, but also NOTCH3 are regulated by mTORC1, HTR2B^+/high^ and NOTCH3^+/high^ CRC cells display overlapping populations with shared common features, and both represent tumor cells with elevated EMT levels.

The CMS subtyping of CRC is of prognostic significance [1]. To enhance its clinical application, various markers have been proposed to distinguish among CMS subgroups, such as HTR2B for CMS4 and CDX2 for CMS2/3 [9]. Interestingly, CDX2 and HTR2B are present in the same tumor samples, showing intratumoral cellular heterogeneity. In another study, different CRC cell lines with CMS1 and CMS4 features were tested in 3D cocultures, and the authors found that CMS4 cells are peripherally localized in the spheroids [26]. We focused on cells with CMS2/3 and CMS4 markers; however, we could not detect such a characteristic distribution. This may be explained by the fact that we focused on other CMS subgroups than CMS1, and that cells studied within the organoids are derived from the same patient in our system. Interestingly, HTR2B also marks a subset of normal epithelial colon cells. High CDX2 and HTR2B levels indicate good and bad patient prognosis, respectively. Thus, there is a heterogeneous expression pattern for these markers within the same patient, and the proportion of CDX2 + and HTR2B + tumor cells may greatly influence patient survival.

EMT has a major impact on the chemo-resistance, metastasis, and recurrence of solid tumors [27] which is partially modulated by epigenetic factors [28]. Increased EMT, fibroblast accumulation, and ECM remodeling are critical hallmarks of CMS4 [1]. Another study highlighted the induction of partial EMT in starved colorectal cancer (CRC) cells [29]. In line with these data, we show that the presence of fibroblasts or collagen-I as ECM induces partial EMT and increases the ratio of HTR2B positive cells in PDOs. Similarly, we also observed an increase in HTR2B + cells in amino acid or carbohydrate-free conditions or with 5-FU treatment. Indeed, a previous study using a co-culture system of CRC cells and fibroblasts found partial EMT and CMS4 gene expression profile in tumor cells in a 3D model [30]. However, the role of fibroblasts and ECM changes have not been separated from each other, and the tumor cell-specific serotonin receptor HTR2B has not been addressed. Thus, our study comprehensively characterizes the HTR2B expression pattern when the niche parameters are changed using the organoid technology.

Human PDAC has increased serotonin levels and expression of key enzymes required for serotonin synthesis. In addition, PDAC cells increase the expression of HTR2B compared to normal pancreatic cells, and stimulating HTR2B allows the better survival of tumor cells in unfavorable conditions [15]. Interestingly, we observed a reduction in the expression of the rate-limiting enzyme TPH1 of 5-HT synthesis in CRC compared to normal colon, and we did not find tumor cells or fibroblasts as major serotonin sources. We observed that the mTOR pathway, known to be induced in the neighbouring surviving cells around dying cancer cells via a paracrine mechanism in unfavorable conditions [23], also induced HTR2B expression, providing a link between a nutrient-deprived environment and the higher HTR2B level. In line with these findings, PDO cells gave a more pronounced response to HTR2B activation under carbohydrate-deprived conditions; however, in contrast to PDAC, serotonin markedly reduced cell viability in CRC. These results suggest that the synthesis and effect of 5-HT is dependent on the cancer type. Since serotonin contributes to the Warburg effect in PDAC cells [15], the contradicting role of 5-HT among different cancer types may originate from the different metabolic adaptation abilities of tumor cells under unfavorable conditions that may also be modified by genetic mutations. The effect of driver mutations on metabolism is illustrated by experiments where the mutation of Apc and KRas in the intestinal epithelium rewired metabolism, increasing glutamine uptake in mice [31].

Serotonin has a dual and opposing function in CRC, inhibiting tumorigenesis in early stages and exerting a tumor-promoting effect in more advanced/metastatic tumors. This has been demonstrated with a colitis-associated carcinoma mouse model whereby HTR2B knockout increased proliferation in early stages. In contrast, in advanced stages of CRC, knocking out HTR2B led to lower proliferation [10]. By using genetically engineered mouse models, Sakita JY et al. reported a novel protective role for 5-HT synthesis that promoted DNA repair activity during the early stages of colorectal carcinogenesis [13, 32]. Our results support the context-dependent effect of serotonin. In contrast to a carbohydrate-free environment, HTR2B activation induced organoid cell migration in collagen-I with no cell death. The migration-inducing effect of 5-HT is also supported by experiments with CRC cell lines by Lee JY et al. [13]. In addition, blocking HTR2B or genetically inactivating this receptor in CRC cell lines in 2D cultures led to reduced migration in another study [13].

Previous studies suggested multiple pathways induced by HTR2B, such as the phosphorylation of Erk1/2 [13], and STAT3 phosphorylation [10]. Although Li T et al. suggested that HTR2B activated the mTOR pathway [14], we found that this pathway also regulates HTR2B expression, raising the possibility of a feedback loop between mTORC1 and HTR2B activity. Interestingly, stimulating HTR2B was published to induce EMT markers via activating the CREB-ZEB1 pathway in CRC [14]. These pathways may all be critical in stimulating EMT and invasion under normal conditions when there is no lack of nutrients. On the other hand, the prolonged HTR2B activation is known to induce mitochondrial dysfunction with decreased ATP synthesis [33]. Thus, we propose that this reduced ATP-producing capability becomes critical only in specific starving conditions, such as the lack of glucose that should serve as an important and direct source for energy production. Still, this effect is negligible when all the nutrients are available for cells. In addition, HTR2B may also interact with other serotonin receptors, such as HTR2A or HTR2C, and with other non-5-HT receptors [34, 35]. These interactions may modify the downstream signalling pathways, including the activation of different G-proteins. Thus, monitoring changes in the 5-HT receptor expression pattern in CRC cells under different conditions and tumor microenvironment (TME) is the next step to uncover the explanation for the dual role of serotonin.

Previous studies co-agreed that HTR2B regulates invasion and tumor cell migration. However, to our knowledge, we are the first to prove the role of serotonin in PDOs, which serve as a better model for human CRC compared to traditional 2D cultures. This also led to the discovery that serotonin induces migration only in collagen, but not in Matrigel when applying 3D conditions. These results highlight the critical role of ECM that is difficult to model in 2D cultures, and they strongly support the use of PDOs when studying human cancers.

In contrast to PDAC, we could not detect serotonin in CRC PDOs and fibroblasts, raising the possibility that serotonin could originate from healthy colonocytes and other surrounding tissues in vivo. For example, colonic enteroendocrine cells are known to be major 5-HT producers in our body [36]. It is also widely reported that amongst patients who take selective serotonin reuptake inhibitors (SSRI) for depression, the risk for CRC development is significantly reduced [37–39], thus evoking the potential of increased serotonin signaling for therapeutic purposes in CRC. SSRIs generally increase the availability of serotonin in the brain and colonic tissue [39] and decrease the concentration in the blood [40]. These data align with our results, showing that serotonin reduces CRC cell viability under some conditions, and it induces migration only when collagen-I accumulates in the ECM, which is an advanced stage of tumorigenesis.

In a mouse model of the mesenchymal CMS4 CRC type with poor survival, Varga J et al. proved that Akt-dependent Notch3 activation drove tumor progression. The authors also found that NOTCH3 expression correlated with CMS4 subgroup and patient survival [6]. Here, we show that HTR2B^+/high^ cells have a higher NOTCH activity compared to HTR2B^−/low^ CRC cells, NOTCH3^+/high^ CRC organoid cells have an enhanced EMT phenotype and a higher HTR2B level, and we also detected the overlap between HTR2B + and NOTCH3 + cells in tumors, suggesting the common features of these two cell populations. Interestingly, we also observed that both NOTCH3 and HTR2B are under the control of mTORC1. This raises a model where mTORC1 activation increases the level of both HTR2B and NOTCH3, explaining why NOTCH3^high^ cells also display a high level of HTR2B. In addition, the NOTCH pathway is induced primarily in NOTCH3^high^/HTR2B^high^ cells by other neighbouring tumor cells and stromal fibroblasts that express NOTCH ligands.

## Conclusions

Collectively, we provide evidence that the ratio of cells with the serotonin receptor HTR2B, previously suggested as a marker of the CMS4 CRC subgroup, is increased under unfavorable conditions, the presence of fibroblasts, and the accumulation of collagen-I. We found that the activation of HTR2B induced migration of tumor cells only in collagen-I. In addition, the higher NOTCH3 level in HTR2B^+/high^ cells and the higher HTR2B expression in the NOTCH3^+/high^ cells indicate these cell populations’ overlapping partial EMT features. Since collagen accumulates in CRC progression, this study not only highlights the context-dependent role of serotonin in CRC tumorigenesis, but it may also provide an explanation for the previous findings on the differential effect of 5-HT in different stages of tumorigenesis. In addition, our results emphasize the critical role of ECM composition when studying features of distinct cell populations.

## Supplementary Information

Supplementary Material 1.

## Data Availability

No datasets were generated or analysed during the current study.
