# Supplementary material for: The aggressive colorectal cancer subtype marker HTR2B has a dual role depending on the tumor microenvironment
Source: Cell Commun Signal. 2025 Oct 1;23:403. doi: 10.1186/s12964-025-02395-6 (PMC12486794; doi:10.1186/s12964-025-02395-6)
Supplement: Supplementary file 1 — Supplementary Material 1. [file 12964_2025_2395_MOESM1_ESM.pdf]

The aggressive colorectal cancer subtype marker HTR2B has a dual role depending on the tumor microenvironment

Idan Carmi, Adrián Orosz, Szabolcs Hajdó, Anikó Zeöld, Tamás Hegedűs, Dóra Kelemen-Győri, Julianna Pozsár, Tamás Tölgyes, Zoltán Wiener

A)

Sample	Gender	Age	Tumor	Differentiation grading	Stage	Nutlin sensitivity	EGF dependency
CRC1	F	63	colon adenocarcinoma	Grade 2	T3N0M0	no	yes
CRC2	M	74	colon adenocarcinoma	Grade 2	T3N0M0	no	yes
CRC3	M	75	colon adenocarcinoma	Grade 2	T3N2aM0	no	yes
CRC4	M	74	colon adenocarcinoma	Grade 2	T3N1M0	yes	yes

B)

Sample	Gender	Age	Tumor	Differentiation grading	Stage
CRC-CAF1	F	39	Colon adenocarcinoma	Grade 2	T3N2bM1a
CRC-CAF2	F	37	Colon adenocarcinoma	Grade 2	T3N1aM0
CRC-CAF4	F	56	Colon adenocarcinoma	Grade 2	T3N1aM0
CRC-CAF6	M	68	Colon adenocarcinoma	Grade 2	T3N0M0
CRC-CAF7	F	55	Colon adenocarcinoma	Grade 2	T3N0M0
CRC-CAF8	M	71	Colon adenocarcinoma	Grade 2	T3N1bM0

C)

Sample #	Gender	Age	Tumor	Differentiation grading	Stage
1	F	54	Rectal adenocarcinoma	Grade 1	pT4b pN0
2	M	75	Colon adenocarcinoma	Grade 1	pT3 pN1c
3	F	75	Colon adenocarcinoma	Grade 1	pT4a pN0
4	F	57	Colon adenocarcinoma	Grade 2	pT3 pN1b
5	F	63	Colon adenocarcinoma	Grade 1	pT3 pN1b
6	M	78	Rectal adenocarcinoma	Grade 3	pT3 pN1a

Table S1. Clinical data of patients. A) PDOs. Nutlin resistance indicates the lack of wild-type *TP53* and EGF dependence marks the lack of mutations in EGF signaling (e.g. KRAS and BRAF). For further characterization of the organoid lines see [1, 2]. B) Clinical data of patients providing RNA for fibroblast analysis. See details in [3]. C) Patient data for immunohistochemical staining of slides and for flow cytometry

Antibody	Producer	Host	Clone/Cat No	Dilution	FC/WM
anti-mouse IgG Alexa 488	Invitrogen	Donkey	A21202	FC 1:100 WM 1:1000 IHC 1:200	FC, IHC and WM
anti-mouse IgG Alexa 568	Invitrogen	Donkey	A10037	FC 1:100 WM 1:1000 IHC 1:200	WM, IHC
anti-rabbit IgG Alexa 488	Invitrogen	Donkey	A21206	FC 1:100 WM 1:1000 IHC 1:200	FC, IHC and WM

anti-rabbit IgG Alexa 568	Invitrogen	Goat	A11011	FC 1:100 WM 1:1000 IHC 1:200	WM, IHC
anti-rabbit IgG Alexa 750	Invitrogen	Goat	A21039	FC 1:100 WM 1:1000	FC
anti-Rat IgG Alexa 488	Invitrogen	donkey	A21208	FC 1:100 WM 1:1000	FC
anti-Rat IgG Alexa 568	Invitrogen	goat	A11077	FC 1:100 WM 1:1000	WM
anti-goat IgG Alexa 568	Invitrogen	donkey	A11057	IHC 1:200	IHC
human CDX2	R&D Systems	Mouse	MAB3665	WM 1:100, IHC 1:100	WM, IHC
human EpCAM PE	BD Biosciences	Mouse	347198	FC 1:100	FC
human FRMD6	Novus	Rabbit	NBP3-05019	WM 1:100	WM
human HTR2B (Ab #3, APC conjugated)	R&D Systems	Rabbit	FAB11297A	FC and sorting 1:100	FC, sorting
human HTR2B (Ab #2)	Novus	Rabbit	NLS1187	FC 1:100	FC
human HTR2B (Ab #1, IC)	Merck	Rabbit	HPA012867	FC 1:100 WM 1:200, IHC 1:200	FC, IHC and WM
human KI67	eBioscience	Mouse	14-5699-82	FC 1:100 WM 1:250	FC and WM
human KI67	abcam	Rabbit	ab16667	FC 1:100 WM 1:250	FC and WM
human lumican	Abcam	Rabbit	ab168348	FC 1:100	FC
human Notch3 Alexa Fluor-488	R&D Systems	Mouse	FAB1559G	FC and sorting 1:100	FC, sorting
human PDGFRA-APC	SONY	Mouse	2217560	FC 1:100	FC
human PDGFRB-PE	SONY	Mouse	2218030	FC 1:100	FC
human Phospho-S6	Cell Signaling	Rabbit	#2211	FC 1:100 WM 1:800	FC and WM
human Vimentin	R&D Systems	Rat	MAB2105	FC 1:100 WM 1:100	FC and WM
human ZEB1	Sigma	Rabbit	HPA027524	WM 1:500	WM
human active caspase-3	R&D Systems	Rabbit	AF835	FC 1:100	FC
human Lumican	R&D Systems	Goat	AF2846	IHC 1:100	IHC
E-cadherin	BD Transduction Laboratories	Mouse	610182	IHC 1:100	IHC

FC: flow cytometry, WM: whole mount immunostaining, IHC: immunostaining of slides, sorting: cell sorting

Table S2. Antibodies used in our studies

Gene	Primer1	Primer2
AXIN2	CTGGCTATGTCTTTGCACCA	CTTCACACTGCGATGCATTT
DDC	TGAGAAAGCTGGAGAAGGGG	TCGGATGAGTAAGCCACCAG
DLL1	CTACTGCAGCTCTTCACCCT	AGGTGCAGGAGAAGTCGTTC
DLL3	CTACCACCGGATGCCTTGTC	GTCCATCTGCACATGTCACC

DLL4	GCGAGAAGAAAGTGGACAGG	GAGCCCATTCTCCAGGTCAT
GAPDH	GGGTGTGAACCATGAGAAGT	CAGTGATGGCATGGACTGTG
HPRT1	TGAGGATTTGGAAAGGGTGT	TCCCCTGTTGACTGGTCATT
HTR2B	TCTGGCTGTTTCACTGGAGA	ATAGAACAAGTGGGAGGGGC
JAG1	CAGAGGCAGCTGTAAGGAGA	CTAACTGGCACGTTTTCCCA
JAG2	GTGTAATTTGCTCCACGGGG	CCAGTTGGTCTCACAGTTGC
KIT	TTCCCCAAACCTGAACACCA	GGCAGCATTGACGTCAGAAT
LGR5	AGTGCTGTGCATTTGGAGTG	AGGGCTTTCAGGTCTTCCTC
MAOA	AATGTGACCTCTGAGCCTCA	AGGTCCATTATCCGTTCGCT
MUC2	ATCCTCAAAAGCAGCGTGTT	CCCCCTCTTTGGTACACTCC
NOTCH1	GCCAACATCCAGGACAACAT	GCGTGTGAGTTGATGAGGTC
NOTCH3	ATGGATGTCAATGTGCGTGG	CAAAGCAGTCTCGCCAGTAC
OLFM4	CAGAGTGGAACGCTTGGAAT	CCTTGATCAGCTCGAAGTCC
PDGFC	TGGTGTGGTTCTGGTACTGT	GGGTAGCACTGAAGGACTCA
PDGFRA	TGTCCCCATGCTAGAAAGGA	TCCTCGGGCAACTTGATAGG
PDGRB	GAAGCAAGCCCTTATGTCGG	AGGAAGGTGTGTTTGTTGCG
SLC18A2	CTGTTCCTTCCGACTGTCCC	GCAGAATCCCGCAAATATGG
SLC6A4	TTCTTCTCTCTTGGTCCGGG	ACCGAGCACTGTGAAGATGA
TPH1	CTCTTAGGTCATGTCCCGCT	AGCCAGCACCAAAGACTCTT
TWIST1	CTGAGCAACAGCGAGGAAG	ACAGCCCGCAGACTTCTTG
ZEB1	GCTGACTGTGAAGGTGTACC	ACATCCTGCTTCATCTGCCT
LUM	CCTGGTTGAGCTGGATCTGT	GTAGGATAATGGCCCCAGGA
VIM	GGTACTCGCATTCTCCACCT	CTCAATGTCAAGGGCCATCT
SNAI1	GATGCACATCCGAAGCCACA	TGACATCTGAGTGGGTCTGG

Table S3. Primers used in our studies

**Neuroendocrine diff genes**	**CMS4 genes in cell lines**	**CMS4 genes**				
Moorman et al	Linnekamp et al	Peters NA et al				
NEUROD1	ABHD17C	ACTA2	CRISPLD1	HOPX	PDE1A	TCEAL7
CHGB	ACSL5	ADAM12	CRYAB	HSD17B6	PDGFC	TGFB1I1
CHGA	CEMIP	ADAMTS12	CTSK	HSPB8	PDLIM3	THBS1
POMC	EHF	AEBP1	CXCL12	HTR2B	PEG3	THBS2
GNAI1	EPCAM	AKAP12	CYP1B1	IGFBP5	PHLDB2	THY1
INSM1	GPR160	ANK2	DCLK1	INHBA	PLN	TIMP2
SCGN	MLLT11	ANTXR1	DCN	ISLR	PPP1R3C	TIMP3
RFX6	NMU	AOC3	DDR2	KCTD12	PRELP	TMEM47
SCG3	NREP	AQP1	DPT	LBH	PRICKLE1	TNC
BTG2	OCIAD2	ARMCX1	DPYSL3	LHFP	PTGIS	TNS1
ITPR2	SEL1L3	ASPN	ECM2	LMOD1	RAB31	TPM2
RAB3C	SERPINB1	BGN	EDNRA	LOX	RARRES2	VCAN
	SH3YL1	BICC1	EFEMP1	LUM	RBMS1	WISP1
	TMC5	BNC2	FBLN1	MAP1B	RBMS3	WWTR1
	TMEM45B	BOC	FBN1	MEIS1	RUNX1T1	ZCCHC24
	TUBA4A	C1R	FBXO32	MFAP2	S1PR3	ZFPM2
		C1S	FERMT2	MGP	SDC2	ZNF521
		C3	FIBIN	MIR100HG	SERPINF1	

CACNA2D1	FLNA	MMP2	SERPING1
CALD1	FN1	MN1	SFRP2
CCDC80	FNDC1	MOXD1	SFRP4
CLDN11	FSTL1	MRGPRF	SGCE
CNN1	FXYD6	MSRB3	SLIT2
COL14A1	GEM	MXRA5	SNAI2
COL15A1	GLT8D2	MYL9	SPARCL1
COL1A1	GPC6	MYLK	SPOCK1
COL1A2	GREM1	NAP1L3	SSPN
COL3A1	GUCY1A3	NDN	STON1
COL5A1	GUCY1B3	NEXN	SULF1
COL5A2	HDGFRP3	PCDH7	TAGLN
COL6A3	HMCN1	PCOLCE	
COL8A1			
COL8A2			
COLEC12			

Table S4. Gene lists used for Fig S3B ([4-6]

**5-FU**	**Org#1**	**Org#2**	**Org#3**	**Org#4**
IC50, nM	18,073	23,603	47,720	36,287

Table S5. IC50 values for 5-FU of the used PDO lines (data from [3]).

Supplementary references

1. Szvicsek Z, Oszvald A, Szabo L, Sandor GO, Kelemen A et al. Extracellular vesicle release from intestinal organoids is modulated by Apc mutation and other colorectal cancer progression factors. Cell Mol Life Sci. 2019;76:2463-2476.

2. Kelemen A, Carmi I, Oszvald A, Lorincz P, Petovari G et al. IFITM1 expression determines extracellular vesicle uptake in colorectal cancer. Cell Mol Life Sci. 2021;78:7009-7024.

3. Soos AA, Kelemen A, Orosz A, Szvicsek Z, Tolgyes T et al. High CD142 Level Marks Tumor-Promoting Fibroblasts with Targeting Potential in Colorectal Cancer. Int J Mol Sci. 2023;24.

4. Linnekamp JF, Hooff SRV, Prasetyanti PR, Kandimalla R, Buikhuisen JY et al. Consensus molecular subtypes of colorectal cancer are recapitulated in in vitro and in vivo models. Cell Death Differ. 2018;25:616-633.

5. Moorman A, Benitez EK, Cambulli F, Jiang Q, Mahmoud A et al. Progressive plasticity during colorectal cancer metastasis. Nature. 2025;637:947-954.

6. Peters NA, Constantinides A, Ubink I, van Kuik J, Bloemendal HJ et al. Consensus molecular subtype 4 (CMS4)-targeted therapy in primary colon cancer: A proof-of-concept study. Front Oncol. 2022;12:969855.

Supplementary Figures

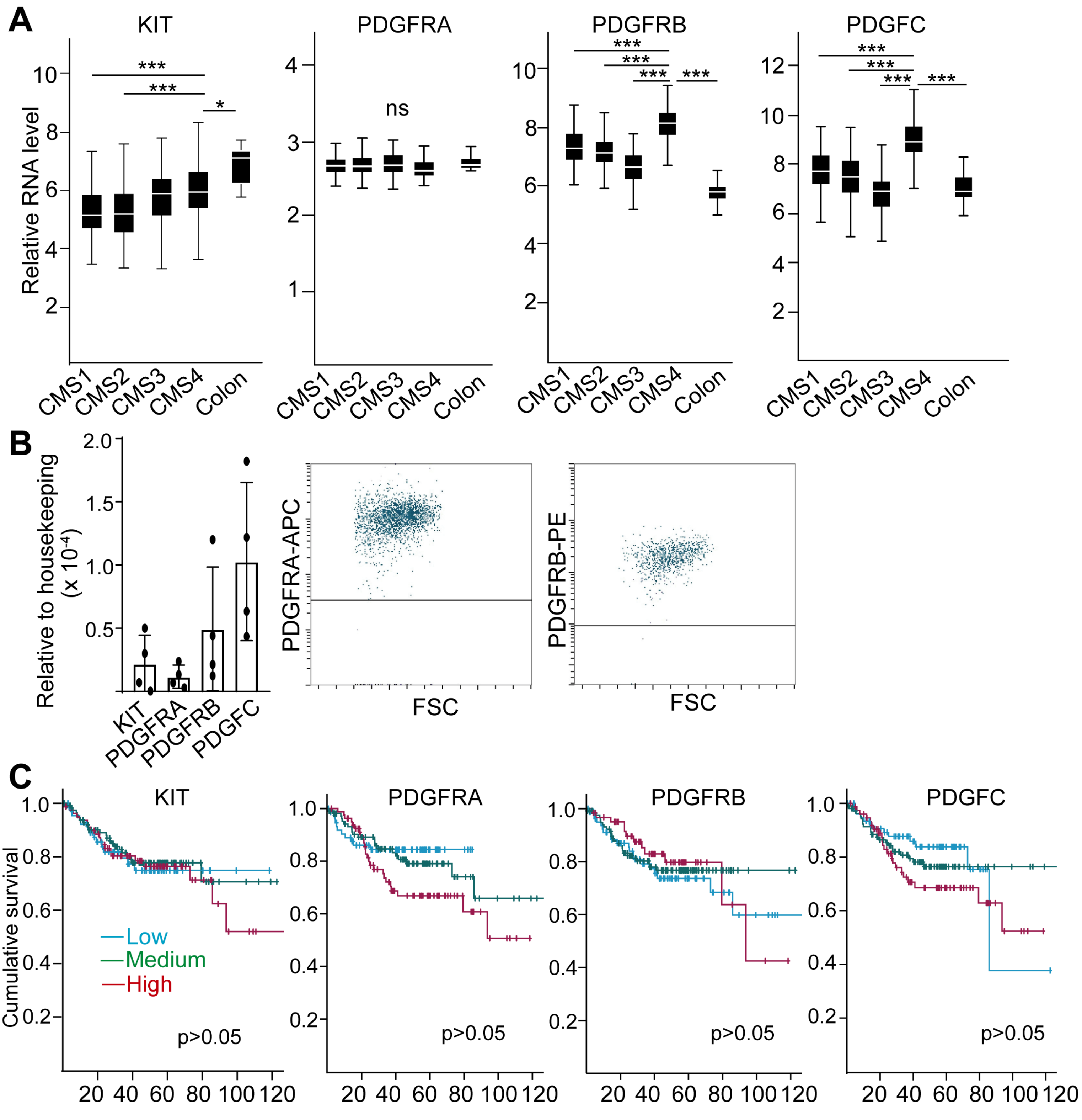

Figure S1. KIT, PDGFRA, PDGFRB, and PDGFC RNA levels do not correlate with patient survival. A) Normalized RNA level of the indicated genes in CMS subgroups and in normal colon samples (analysis of the GSE39582 GEO dataset). B) KIT, PDGFRA, PDGFRB, PDGFC RNAs in PDOs (RT-qPCR), and representative flow cytometry dot plots for PDGFRA and PDGFRB from one organoid line. C) Kaplan-Meier plot for the indicated genes (analysis of the GSE17537 and GSE14333 data sets, low expression group: z-score normalized data <-0.5*SD, high expression: z-score normalized value >0.5*SD, medium: -0.5*SD<z-score normalized data<0.5*SD). p-values from the log-rank test are shown.

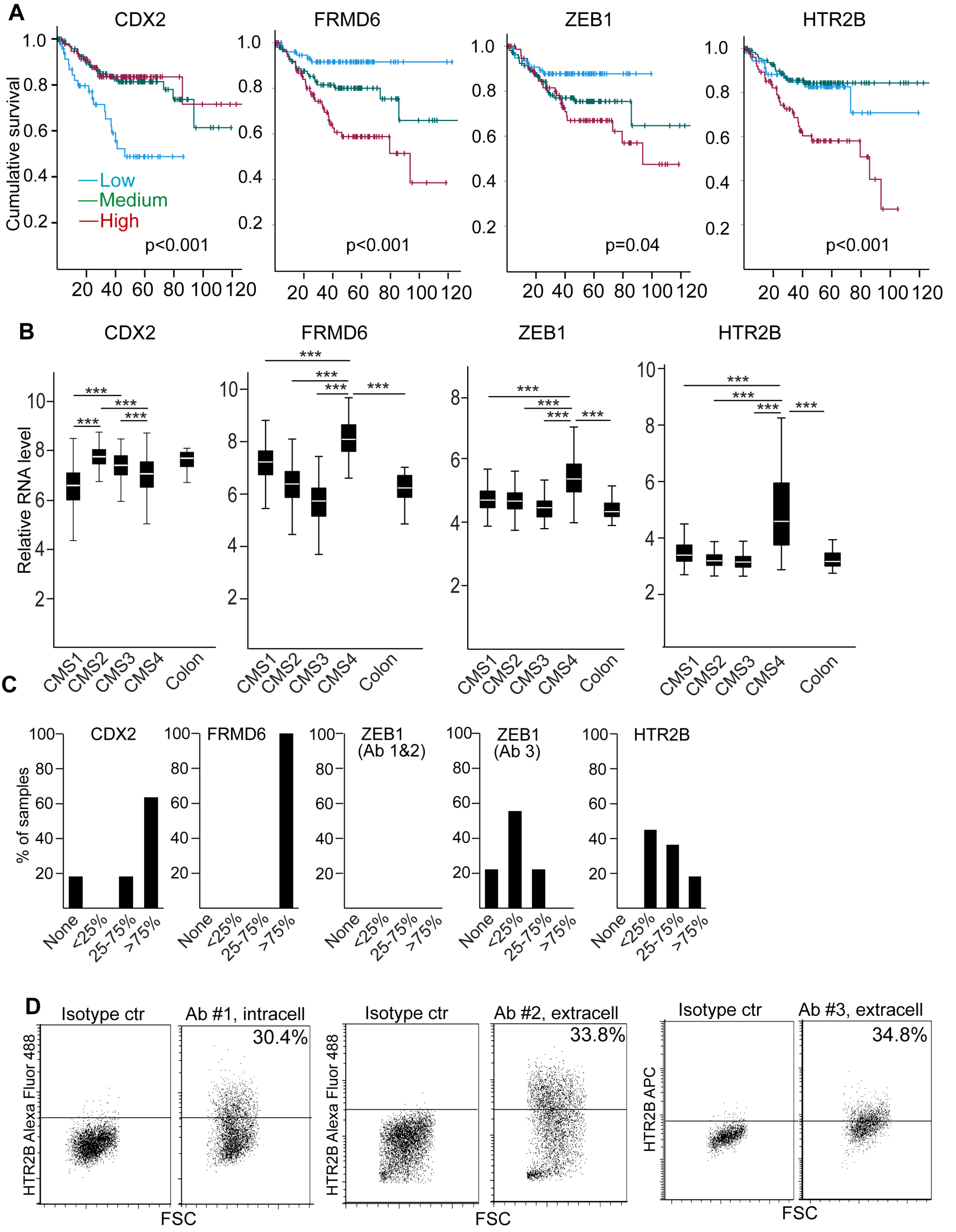

A
CDX2
FRMD6
ZEB1
HTR2B
Cumulative survival
Low
Medium
High
p<0.001
p<0.001
p=0.04
p<0.001
B
CDX2
FRMD6
ZEB1
HTR2B
Relative RNA level
***
CMS1
CMS2
CMS3
CMS4
Colon
C
CDX2
FRMD6
ZEB1 (Ab 1&2)
ZEB1 (Ab 3)
HTR2B
% of samples
None
<25%
25-75%
>75%
D
Isotype ctr
Ab #1, intracell
30.4%
Isotype ctr
Ab #2, extracell
33.8%
Isotype ctr
Ab #3, extracell
34.8%
HTR2B Alexa Fluor 488
HTR2B APC
FSC

Figure S2. Analysis of the expression pattern of CMS2/3 (CDX2) and CMS4 (ZEB1, FRMD6, HTR2B) markers. A) Kaplan-Meier survival plot for CDX2, FRMD6, ZEB1, and HTR2B (low expression group: z-score normalized data <-0.5*SD, high expression: z-score normalized value >0.5*SD, medium: -0.5*SD<z-score normalized data<0.5*SD). p-values from the log-rank test are shown. B) Normalized RNA level of the indicated genes in CMS subgroups and in normal colon samples (GSE39582 GEO dataset). C) Analysis of tissue sections for the indicated markers from the Protein Atlas database. The quantity values were processed that are available for 12 samples with each antibody (for ZEB1, antibody1: HPA027524, antibody2: CAB058686, antibody3: CAB079943). The horizontal axis shows the percentage of positive cells within a single tissue section, and the y-axis refers to the ratio of samples falling into the respective category of the x-axis. D) Flow cytometry of a PDO line for three different anti-HTR2B antibodies. The horizontal line marks the isotype or secondary antibody control for each measurement.

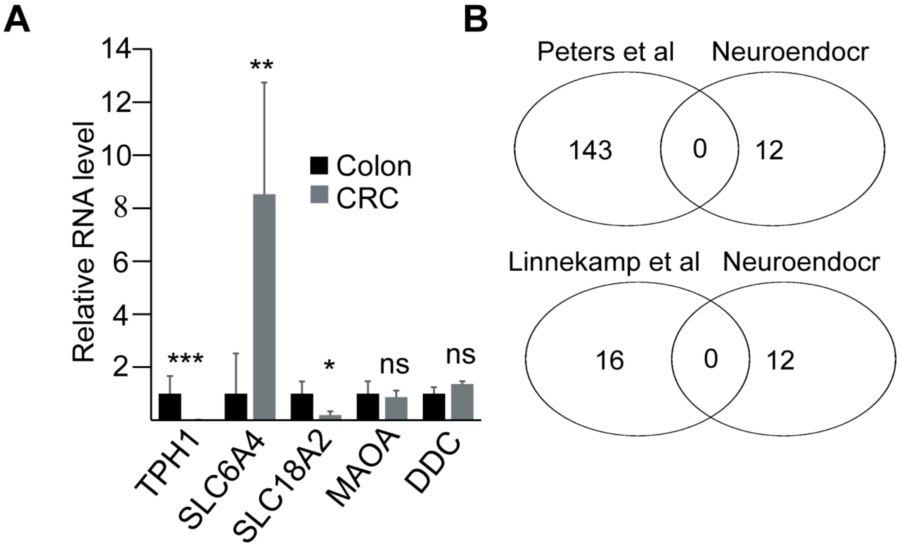

Figure S3. CRC organoids express the rate-limiting enzyme of serotonin synthesis at a reduced level compared to normal colon. A) RT-qPCR analysis of the indicated genes. Housekeeping normalized expression values were compared to data from normal colon organoids (n=4-4, unpaired colon and CRC organoid samples). Unpaired t-test, ***p<0.005, **p<0.01, *p<0.05, ns: p>0.05. B) Overlap between the indicated gene sets with the number of genes in each category. For details, see Table S4

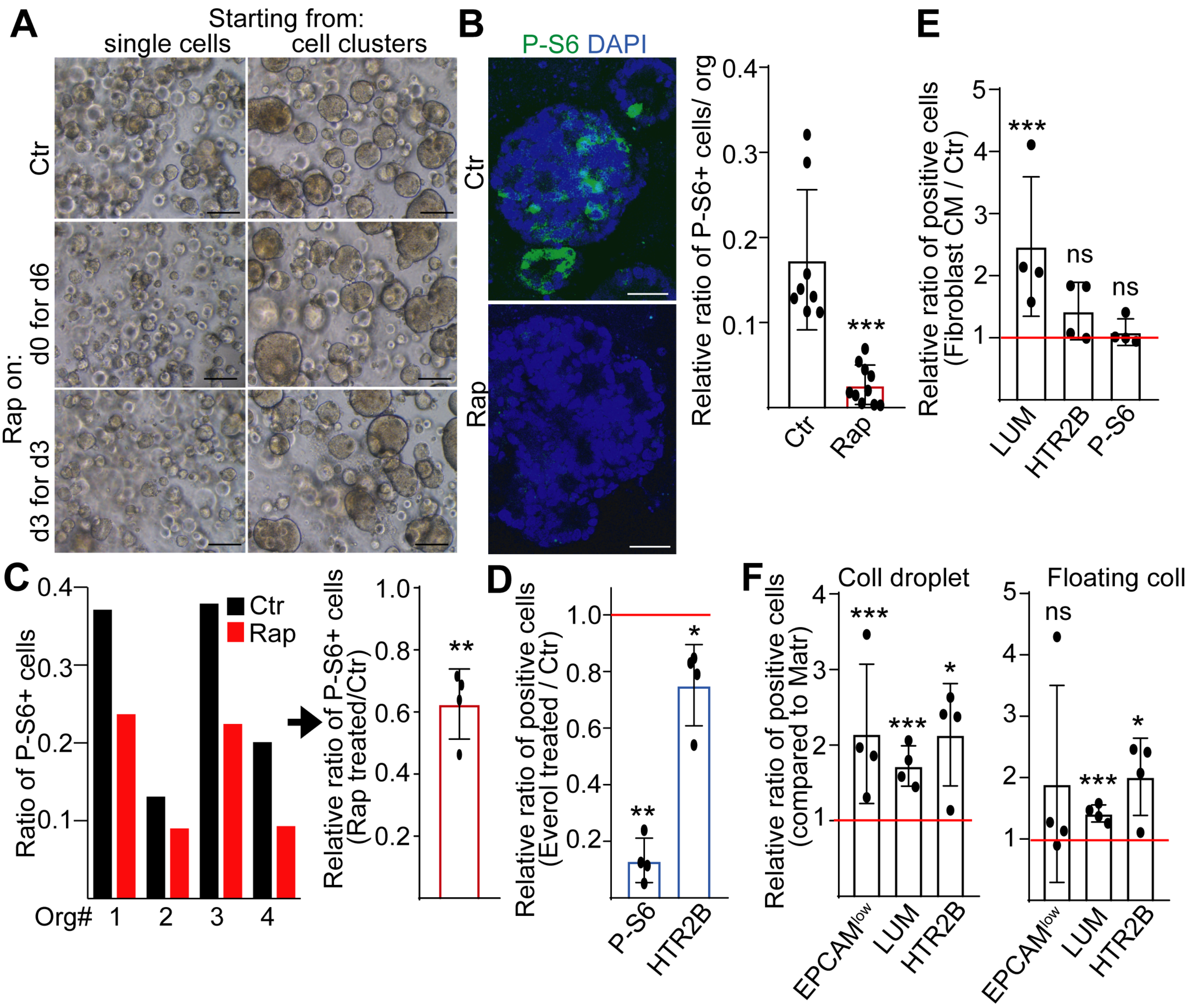

Figure S4. Different factors modulating the ratio of cells with phospho-S6 or HTR2B. A) Light microscopy images from a PDO culture (#1) with or without rapamycin (mTORC1 inhibitor, 10 μM) treatment on day 6. Rapamycin was added either directly after splitting the organoids for 6 days or only on day 3 of culturing for 3 days. Cultures were started either from single cells or from cell clusters. Note the lack of major morphological effects of rapamycin. B) Ratio of phospho-S6+ (mTORC1 target) cells within the organoids in the presence or absence of rapamycin, applied for 2 days (representative confocal microscopy images and their quantification, 6 images from PDO line #1). C) Ratio of P-S6+ cells with or without rapamycin (for 2 days), measured by flow cytometry. The left panel shows individual PDO lines, and the right panel shows their mean and SD. Note the heterogeneity among PDO lines. D) Ratio of P-S6+ or HTR2B+ cells in the presence of everolimus (mTORC1 inhibitor, 1μM for 2 days) compared to the untreated control (red line). E) Changes in the ratio of positive cells for the indicated markers when cultured in fibroblast derived conditioned medium for 2 days. Confluent fibroblast cultures were maintained in CRC organoid medium for 2 days before harvesting conditioned medium. The red line indicates control PDOs. F) Changes in the ratio of positive cells for EPCAM^{low}, LUM or HTR2B in collagen adherent droplets or in floating collagen gel. Data were compared to Matrigel control. For defining the EpCAM^{low} population, see Fig 3C. n=4 (C, D, E, F). 8-10 organoids were evaluated from PDO line #1 for B). Scale bars: 50μm (A), 20μm (B). Unpaired (B) or paired (C-F) t-tests were applied with ***p<0.005, **p<0.01, *p<0.05, ns: p>0.05

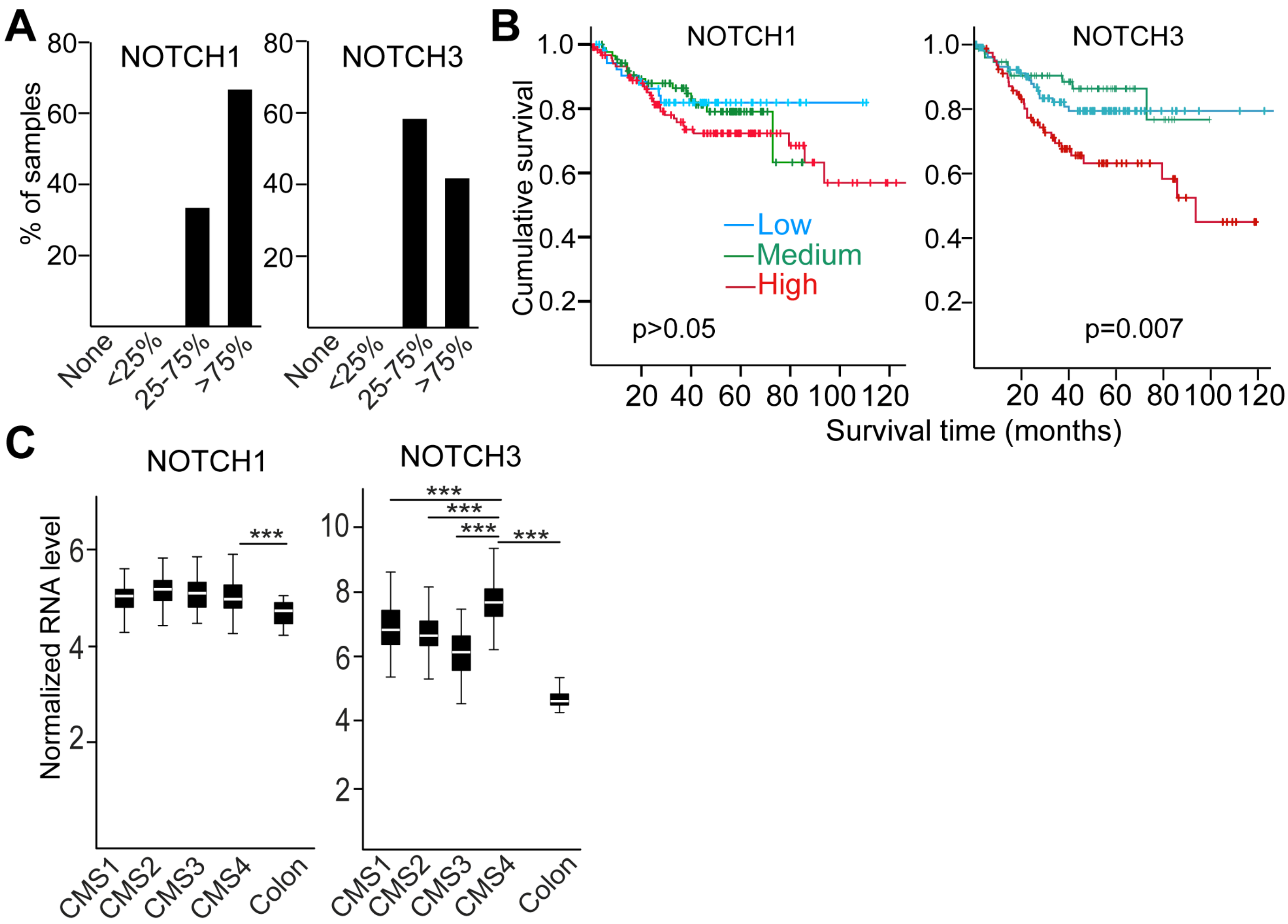

Figure S5. The expression patterns of NOTCH1 and NOTCH3 and their correlation with patient survival. A) Analysis of tissue sections for NOTCH1 and NOTCH3 from the Protein Atlas database. The quantity values were processed that are available for 12 samples with each antibody. B) Kaplan-Meier survival plot for NOTCH1 and NOTCH3. p-values from the log-rank test are shown. Note the lack of significant difference for NOTCH1. C) Normalized RNA levels of NOTCH1 and NOTCH3 in CMS subgroups and in normal colon samples.
